# Green Synthesis of Cellulose Acetate Mixed Matrix Membranes: Structure–Function Characterization

**DOI:** 10.1021/acssuschemeng.4c07538

**Published:** 2025-01-16

**Authors:** Andrea Torre-Celeizabal, Francesca Russo, Francesco Galiano, Alberto Figoli, Clara Casado-Coterillo, Aurora Garea

**Affiliations:** † Department of Chemical and Biomolecular Engineering, Universidad de Cantabria, Av. Los Castros s/n, 39005 Santander, Spain; ‡ Institute on Membrane Technology (CNR-ITM), Via P. Bucci 17/C, 87036 Rende (CS), Italy

**Keywords:** cellulose acetate, dimethyl carbonate, green solvents, membrane characterization, gas permeation characterization

## Abstract

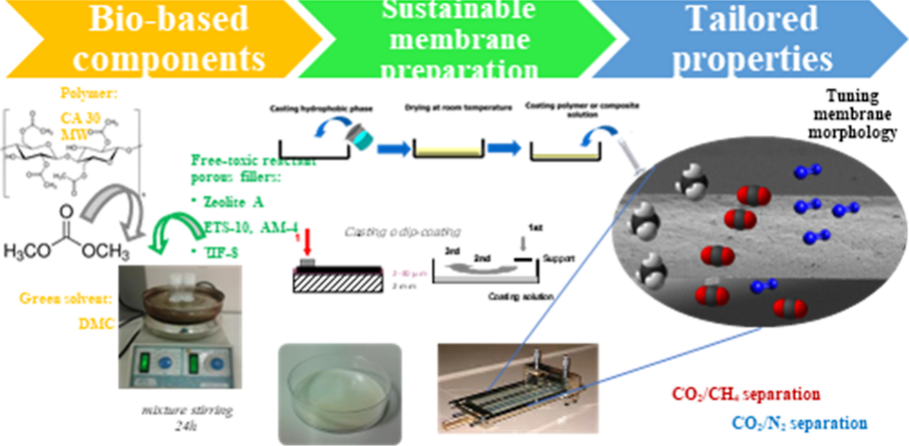

Although membrane technology is widely used in different gas separation applications, membrane manufacturers need to reduce the environmental impact during the membrane fabrication process within the framework of the circular economy by replacing toxic solvents, oil-based polymers, and such by more sustainable alternatives. These include environmentally friendly materials, such as biopolymers, green solvents, and surfactant free porous fillers. This work promotes the use of environmentally sustainable and low toxic alternatives, introducing the novel application of cellulose acetate (CA) as a biopolymer in combination with dimethyl carbonate (DMC) as a greener solvent and different inorganic fillers (Zeolite-A, ETS-10, AM-4 and ZIF-8) prepared without the use of toxic solvents or reactants. Hansen Solubility Parameters were used to confirm the polymer–solvent affinity. Pure CA and mixed matrix membranes were characterized regarding their hydrophilicity by water uptake and contact angle measurements, thermal stability by TGA, mechanical resistance, ATR-FTIR and scanning electron microscopy before evaluating the gas separation performance by single gas permeability of N_2_, CH_4_, and CO_2_. Conditioning of the CA membranes is observed causing reduction of the CO_2_ permeability values from 12,600 Barrer for the fresh 0.5 wt % ETS-10/CA membrane to 740 Barrer for the 0.5 wt % ZIF-8/CA membranes, corresponding to 24% and 4.2% reductions in CO_2_/CH_4_ selectivity and 30% and 24% increase in CO_2_/N_2_ selectivity for the same membranes. The structure–relationship was evaluated by phenomenological models which are useful at low filler loading considering flux direction and particle shape and size but still fail to explain the interactions between the DMC green solvent and CA matrix and fillers that are influencing gas transport performance different than other CA membranes.

## Abstract 2

This manuscript addresses important issues on the fabrication of membranes with components selected with sustainability criteria: biopolymers, fillers and green solvent, and the correlation of physicochemical and morphological properties with gas permeation performance.

## 1 Introduction

Following the recommendation of the Sixth Assessment Report of the Intergovernmental Panel on Climate Change,^1^ biogas upgrading technologies are being developed to reduce CO_2_ emissions to the atmosphere and meet the world energy demand.^2,3^ Membrane technology features a modest energy consumption, easy processability, high flexibility, easy maintenance, low cost and environmental footprint that make membranes a sustainable approach compared to other biogas upgrading technologies.^4−7^ The actual interest in returning to the circular economy focuses the stress on membrane fabrication using renewable rather than petrochemical-based or toxic materials. Biopolymers are defined as polymers coming from renewable sources that provide several benefits such as biodegradability, biocompatibility, low cost, easy processability and safe waste disposal.^8−10^ Recently reports stress the potential of different biopolymers, such as chitosan (CS),^11^ poly lactic acid,^12^ poly(vinyl) alcohol (PVA),^13^ polyurethane (PU),^14^ starch^12^ and cellulose acetate (CA)^15^ in gas separation processes. CA is the most abundant natural polymer, and CA based membranes have been commercialized for more than 30 years.^16^ The permeability, selectivity, and stability are still below other nonrenewable polymer membranes in decarbonization applications needing to treat large volumes of gas. This is due to the effect of high CO_2_ partial pressure, conditioning and physical aging of CA, which cause swelling and segmental mobility increases of the polymeric chains, ultimately altering the permeability and selectivity over the long-term.

Generally, the low CO_2_ separation performance of pure CA membranes has been attempted to increase by different modifications. First, the control of deacetylation degree of CA has been extensively studied showing minor changes in crystallinity and increased permeability and hydrophilicity of the membranes without undermining their thermal or mechanical stability.^19^ Abdellah et al. observed that a decrease in deacetylation degree caused an increased in hydrophilicity while the glass transition and thermal stability of the CA membranes was maintained.^33^ A very widespread modification to improve the gas separation performance of glassy polymers are mixed matrix membranes (MMM), consisting on the loading of a small amount of organic or inorganic particle fillers into the continuous polymer matrix to obtain a new hybrid heterogeneous material with synergic properties.^34^ Zeolites and zeotype porous materials have been the most used fillers. Metal–organic frameworks (MOFs) are considered a good choice as well because their organic nature allows the expectation of good compatibility with the polymer matrix to prepare defect-free membranes. To cite some of the examples collected in Table 1, Mubashir et al. observed an increase in CO_2_ permeability from 15.8 to 84.8 Barrer, while CO_2_/CH_4_ selectivity varied from 12.2 to 35.3 when loading CA with 8 wt % of ZIF-62.^30^ Alkandari et al. incorporated 10 wt % ZIF-67 in CA achieving a CO_2_/CH_4_ selectivity of 16.16 with a CO_2_ permeability of 17.29 Barrer.^31^ Tanvidkar et al. used multiwalled carbon nanotubes (MWCNTs) with UiO-66-NH_2_ and observed an increase in permeability with similar selectivity values as the pristine CA polymer.^26^

**Table 1:** Review of Literature on CA-Based Membranes for Gas Separation^a^

polymer continuous phase	solvent	filler dispersed phase	physicochemical characterization techniques	performance test conditions	gas permeability (Barrer)	reference
CA with DS = 1.75, 2.45 and 2.84	acetone	none	DSC, TGA, WAXS, mechanical tests, gas sorption	pure gas permeability, 35 °C, 1 atm	*P*(CO_2_) = 4.75 *P*(N_2_) = 0.15 *P*(CH_4_) = 0.15	Puleo et al.^16^
CA (Eastman)	EtOH, THF at solvent ratios of 4 and 2	none	FE-SEM, mechanical tests, TGA	pure and mixed gas permeation (40:50 v/v % CO_2_/CH_4_), 40 °C, *p* = 0.7 MPa	*P*(CO_2_) = 4.75^b^*P*(N_2_) = 0.5^b^*P*(CH_4_) = 0.6^b^	Pak et al.^17^
CA (acetyl content: 54.6–56%)	acetic acid/water (60:40, 70:30, 80:20)	none	ATR-FTIR	pure gas permeability, ambient temperature	*P*(CO_2_) = 400.93^b^*P*(N_2_) = 12.18^b^	Jawad et al.^18^
CDA and CTA	NMP	none	FTIR, XRD, SEM, mechanical tests	pure gas permeation, 25 ± 1 °C, *p* = 5 bar	*P*(CO_2_) = 17.32 *P*(CH_4_) = 0.93	Raza, Farrukh et al.^19^
CA	THF	zeolite NaY	ATR-FTIR, SEM	pure gas permeation, 25 °C, 4–22 bar	*P*(CO_2_) = 4.87 *P*(N_2_) = 0.2	Sanaeepur et al.^20^
CA (acetyl content: 54.6–56%)	acetic acid/water (70:30)	MWCNTs	TEM, Raman module, FESEM, XRD, viscosity measurements, mechanical tests	pure gas permeation, feed 100 mL/min, room temperature, 1–3 × 10^5^ Pa	*P*(CO_2_) = 830 *P*(N_2_) = 96.06	Ahmad et al.^21^
CA (MW = 50,000, Sigma-Aldrich)	THF	TiO_2_	SEM, FTIR, mechanical tests	pure gas permeation, 25 °C, 1–5 bar	*P*(CO_2_) = 26 *P*(N_2_) = 19 *P*(CH_4_) = 56	Rashid et al.^22^
CA (MW = 50,000, Sigma-Aldrich)	THF	MgO	FESEM, EDS, XRD, FTIR, TGA, DSC, BET	pure gas permeation, ambient temperature, 1–4 bar	*P*(CO_2_) = 62.90 *P*(CH_4_) = 2.59	Rajpure et al.^23^
CA (acetyl content: 39.7%; MW = 50,000, Sigma-Aldrich)	DCM	P[CA-Im][Tf_2_N] ionic liquid (IL)	NMR, FTIR, DSC, TGA, SEM	mixed gas permeation (15:85% to 85:15% CO_2_/N_2_), 26 °C, 5 bar	*P*(CO_2_) = 1890 *P*(N_2_) = 71.3	Nikolaeva et al.^24^
CA (purity >99.99%, MW = 28,000)	NMP	UiO-66-NH_2_	XRD, FTIR, SEM, EDS, BET, mechanical tests, TGA	pure gas permeation, 25 °C, 0.3 MPa	*P*(CO_2_) = 168.8 *P*(N_2_) = 4.2 *P*(CH_4_) = 3.3	Hu, Miu et al.^25^
CA	DMF	UiO-66-NH_2_/MWCNTs	FTIR, XRD, TGA, DSC, FESEM	pure and mixed gas permeation (40:60% CO_2_/CH_4_), 30 °C, 1.5 bar	*P*(CO_2_) = 31.65 *P*(CH_4_) = 1.89	Tanvidkar, Nayak et al.^26^
CA	NMP	ZIF-8	XRD, SEM, FTIR, EDS, BET, TGA, DSC	pure gas permeation, 25 °C, 0.3 MPa	*P*(CO_2_) = 170.2 *P*(CH_4_) = 7.6	Hu, Zhang et al.^27^
CA	DMF	ZIF-8	XRD, TGA, DSC, FTIR, FESEM	pure and mixed gas permeation (40:60% CO_2_/CH_4_), 30 °C, 1.5 bar	*P*(CO_2_) = 9.65 *P*(CH_4_) = 0.93	Tanvidkar, Jonnalagedda et al.^28^
CA (acetyl content: 39.7%)	DMF	ZTIF-1	SEM, EDS, FTIR, XRD, TGA, DSC mechanical tests	pure gas permeation, 35 °C, 105 kPa	*P*(CO_2_) = 15.85 *P*(N_2_) = 0.73 *P*(CH_4_) = 0.81	Li et al.^29^
CA	NMP	ZIF-62	BET, SEM, XRD, FTIR, NMR, TGA	mixed gas permeation (50:50% CO_2_/CH_4_), feed 300 mL/min, 25 °C, 3 bar	*P*(CO_2_) = 84.8 *P*(CH_4_) = 2.40	Mubashir et al.^30^
CA	acetone and DMAc (2:1 v/v)	ZIF-67	XRD, TGA, FTIR, SEM–EDX, gas sorption	pure gas permeation, 25 °C, 1 bar	*P*(CO_2_) = 17.29 *P*(N_2_) = 1.02 *P*(CH_4_) = 1.07	Alkandari et al.^31^
CTA (DS = 2.87) and CDA (DS = 2.4) (Eastman, USA)	NMP	NH_2_-ZIF-8 (15 wt %)	XRD, FESEM, FTIR, TGA-DTA	pure gas at 35 °C, 5 bar, mixed gas at 10 bar and 35 °C, binary CO_2_/CH_4_ (50:50, v/v)	*P*(CO_2_) = 11.33 *P*(CH_4_) = 0.34	Raza, Japip et al.^32^

a BET: Brunauer Emmett and Teller surface area; CA: cellulose acetate; CDA: cellulose diacetate; CTA: cellulose triacetate; DS: degree of substitution; DCM: dichloromethane; DMAc: *N*,*N*,dimethylacetamide; DMF: dimethylformamide; DSC: differential scanning calorimetry; EDS: energy dispersive spectrum; EDX: energy-dispersive X-ray spectroscopy; EtOH: ethanol; FESEM: field emission scanning electron microscopy (SEM); FTIR: Fourier transform infrared spectroscopy; IL: ionic liquids; MgO: magnesium oxide; MWCNTs: multiwalled carbon nanotubes; NMP: *N*-methyl-2-pyrrolidone; NMR: nuclear magnetic resonance spectroscopy; SEM: scanning electron microscopy; TEM: transmission electron microscopy; TGA: thermal gravimetric analyses; THF: tetrahydrofuran; WAXS: wide-angle X-ray scattering; WHC: water holding capacity; XPS: X-ray photoelectron spectroscopy; XRD: X-ray diffraction; ZIF: zeolitic imidazolate framework; ZnO: zinc oxide; ZTIF-1: zeolitic tetrazolate-imidazolate framework.

b This value is given as permeance in GPU.

Recent approaches, however, are focused on improving the sustainability of CA membrane fabrication as well, as the synthesis of bio-CA membranes proposed by Khamwichit et al. using CA produced from coconut juice waste.^35^ Additionally, as also shown in Table 1, the most common organic solvents used for the preparation of CA membranes are *N*,*N*-dimethylacetamide (DMAc), *N*-methyl pyrrolidone, *N*,*N*-dimethylformamide (DMF), tetrahydrofuran (THF), acetone, and propanol, all of which have high environmental and health impacts.^8,15^ Green solvents are organic molecules derived from renewable and recyclable resources designed to minimize the environmental footprint associated with their use. In addition to their low toxicity and reduced risk to human health and the environment, other factors are evaluated to classify a solvent as green. These include the environmental impacts from their production, use, and disposal, such as the depletion of nonrenewable resources, potential for solvent recycling, and energy consumption involved in their synthesis, recycling, and waste management. For this aim, green solvents are attracting increasing interest for sustainable membrane fabrication.^36^ However, the use of this kind of solvents in membrane technology is still in early stages.^9^ Wang et al. used PolarClean as a greener solvent for membrane preparation via nonsolvent-induced phase separation and obtained that the performance of the prepared membranes was competitive with the state-of-the-art membranes in water treatment.^37^ Russo et al. prepared polyether sulfone (PES) and poly(vinylidene fluoride) (PVDF) membranes by using dimethyl isosorbide as greener solvent for ultrafiltration and microfiltration and demonstrated that these membranes gave suitable results for these applications.^38^ Tomietto et al. combined a microbial biopolymer with biocompatibility, high resistance, and biodegradability, polyhydroxyalkanoate (PHA), with the biodegradable, nontoxic, and renewable green solvent Cyrene, and obtained dense membranes that were successfully applied in pervaporation.^10^ Nevertheless, one of the main properties of CA is the ability to be dissolved in green solvents, as for example methyl lactate.^39^ The fabrication of well-dispersed homogeneous MMMs is slightly influenced by the polymer/solvent system. Hansen solubility parameters (HSPs) may provide insight of the distance between the solvent and the polymer considering the dispersive (δ_d_), polar (δ_p_), and hydrogen bond forces (δ_H_), and this can be used to predict the solubility and compatibility of a polymer in different solvents that will result in a homogeneous solution.^9,38,40^ In particular, the dispersive forces (δ_d_) is represented by nonpolar interactions which are common among hydrocarbon chains and contribute to the general stability of the molecular structure; the polar forces (δ_p_) is based on the dipole–dipole interactions, which are important for understanding compatibility in systems where polarity influences solubility or miscibility, particularly in solvents with partial charges, and hydrogen bonding (δ_h_) significantly influenced the solubility of polymer.

Most recently, HSPs have been highlighted as a tool to evaluate the compatibility between polymers and fillers to overcome the adhesion issue in the preparation of MMMs^41,42^ and ensuring compatibility and stability in membrane fabrication. Their predictive capacity has supported the design of membranes, paving the way for optimal performance.

Only a few researchers have yet used green solvents for the fabrication of gas separation membranes. Bridge et al. used Cyrene, a glucose-based polar aprotic solvent, to produce defect-free membranes with polysulfone (PSf) and obtained H_2_ permeances of more than 100 GPU.^43^ Papchenko et al. compared the use of chloroform, as the commonly used solvent, and dimethyl carbonate (DMC), as the green solvent, with low toxicity to prepare membranes using poly(hydroxybutyrate-*co*-hydroxyvalerate) (PHBV).^44^ Their results indicated that gas permeability was very similar for both membranes as well as their physical and chemical properties, independently of the solvent employed for the membrane preparation. Less viscous green solvents than Cyrene or PolarClean have been used to dissolve CA, as methyl lactate, methyl tetrahydrofuran, DMC, or triethylphosphate but always for making porous filtration membranes.^33,40^ The most common way to introduce nanoporosity to improve simultaneously the permeability, selectivity, and mechanical endurance of polymer membranes are MMMs, which consist in a small amount of loading of a porous inorganic filler into the polymer matrix to create a new hybrid defect-free material with synergistic properties. The morphology, chemical composition, and size of the particles are important to interact with the polymer chains and make defect-free membranes. The addition of small loadings of porous surfactant-free titanosilicate materials, of 2D and 3D structures, as ETS-10 and AM-4,^45,46^ respectively, a commercial zeolite 4A^47^ and zeolite imidazolate framework ZIF-8,^27^ which have an affinity for CO_2_, in very small amounts, allowed the study of their effect in CA-DMC membranes without compromising the processability of the pure biopolymer membranes.

This work aims to go one step further in promoting sustainability through the use of green solvents in gas separation applications. CA membranes were prepared using DMC as a green solvent and loaded with selected porous fillers of different morphology compositions and particle size. The affinity between these materials was first initially assessed and confirmed through HSPs. Gas separation performance was measured in terms of pure N_2_, CH_4_, and CO_2_ permeabilities. The structure-performance relationship of the membranes was completed by using different characterization techniques, such as water contact angle (WCA), mechanical tests, thermogravimetric analysis (TGA), SEM, and attenuated total reflectance Fourier transformed infrared spectroscopy (ATR-FTIR).

## 2 Experimental Materials and Methods

### 2.1 Materials

The polymer used for the fabrication of the membranes was CA (average *M*_n_ ∼ 50,000, Sigma-Aldrich, Spain). The green solvent was DMC (Merk, Spain). The commercial nanoporous fillers used were Zeolite NaA (Molecular sieves, 4A. Sigma-Aldrich, Spain) and ZIF-8 (Basolite Z1200, Sigma-Aldrich, Spain).

### 2.2 Hansen Solubility Parameters

HSPs were applied to describe the total energy of vaporization of the components considering the different molecular interactions between them, from the dispersive forces (δ_d_), polar forces (δ_p_), and hydrogen bonding (δ_h_), parameters that are specific of each material as well reported in the literature.^48^

In this way, the Hildebrand solubility parameter (δ_T_) in eq 1 represents the general solubility considering the cohesive energy of HSPs
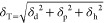
1

This parameter provides information about the solubility capability of a polymer in a particular solvent. The closer the δ parameters are the greater the expected solubility.^38^ On the other hand, the similarity between two components of a system pair (polymer–solvent, polymer-penetrant gas, polymer–filler) can be expressed as the distance in the Hansen space
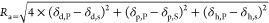
2

### 2.3 Membrane Preparation

Flat-sheet CA membranes were prepared using DMC as solvent by a solution casting method. Table 2 summarizes the membranes prepared in this work. In a typical synthesis, a dope solution of CA (5 wt %) was stirred in DMC overnight to ensure complete polymer dissolution. Once a clear solution was obtained, it was left for 2 h without stirring before the casting to remove any air bubbles. Later, the solution was poured in a glass Petri dish, and the solvent was evaporated in a fume-hood at ambient temperature for 2 days. Subsequently, the membrane was separated from the Petri dish by immersion in a deionized water bath at room temperature and washed twice to remove the excess of solvent. MMM were prepared by adding different types of fillers previously used in our research group, such as 3D Zeolite 4A, 3D ETS-10 titanosilicate, 2D AM-4 titanosilicate, and the MOF, ZIF-8. The lamellar titanosilicate fillers used in this work were previously prepared by hydrothermal synthesis without organic surfactant-directing structural agents as reported elsewhere.^46,49^ All the fillers were dispersed in 2 mL of the solvent under stirring for 24 h and sonicated for 40 min before being added to the polymeric solution in a 0.5, 1, and 2.5 wt % loading content, with respect to the polymer content, according to
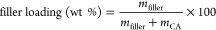
3where *m*_filler_ and *m*_CA_ are the weight of the respective filler particles and CA, respectively, in the membrane casting solution. The DMC solvent was evaporated at room temperature for 2–3 days and stored before characterization without further treatment.

**Table 2:** List of the CA-DMC Membranes Prepared and Characterized in This Work

membrane code	polymer	solvent	filler	wt % polymer	wt % filler
CA	CA	DMC		5	
0.5 wt % zeolite A/CA	CA	DMC	zeolite-4A	5	0.5
1 wt % zeolite A/CA	CA	DMC	zeolite-4A	5	1
2.5 wt % zeolite A/CA	CA	DMC	zeolite-4A	5	2.5
0.5 wt % ETS-10/CA	CA	DMC	ETS-10	5	0.5
1 wt % ETS-10/CA	CA	DMC	ETS-10	5	1
2.5 wt % ETS-10/CA	CA	DMC	ETS-10	5	2.5
0.5 wt % AM-4/CA	CA	DMC	AM-4	5	0.5
1 wt % AM-4/CA	CA	DMC	AM-4	5	1
2.5 wt % AM-4/CA	CA	DMC	AM-4	5	2.5
0.5 wt % ZIF-8/CA	CA	DMC	ZIF-8	5	0.5
1 wt % ZIF-8/CA	CA	DMC	ZIF-8	5	1
2.5 wt % ZIF-8/CA	CA	DMC	ZIF-8	5	2.5

### 2.4 Membrane Characterization

Membrane thickness was measured by a Digimatic micrometer (Mitutoyo 543–561D, Metric Dial indicator, 0 → 30 mm measurement range, 0.0005 mm, 0.001 mm resolution, 1.5 μm, Japan). The average thickness and standard deviation were calculated from the measurements in five different regions of the membrane. The morphological analysis of the membranes was performed by using a scanning electron microscope (Zeiss EVO, MA100, Assing, Italy). The samples for cross sections were obtained by freeze-fracturing them in liquid nitrogen and sputtered with a thin gold layer before analysis.^50^

The water uptake (WU) of the membrane was calculated by eq 4 as
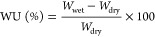
4where *W*_dry_ is the weight of the dry membrane and *W*_wet_ the weight of the membrane soaked in distilled water for 24 h. Both values were averaged from three pieces of each membrane sample to ensure reproducibility.

The wettability of the membrane was estimated by using the sessile drop method (CAM200 Instrument, KSV Instruments LTD, Helsinki, Finland) using ultrapure water (5 μL). The measurements of each membrane were acquired, and the average, as well as the standard deviation, was calculated.

The mechanical strength of the membranes was measured with a Zwick/Roell Z 2.5 (Ulm, Germany) instrument to obtain the Young modulus and the elongation at break, which indicates the resistance to deformation as well as the elasticity of the membrane. The procedure consists on stretching the sample unidirectionally at a constant velocity of 5 mm min^–1^. Each membrane was measured three times.

The ATR-FTIR analyses of the membranes were performed in a PerkinElmer spectrometer. This technique allows us to observe the chemical composition of the membrane surface and estimate the functional groups as well as the interaction between them. The spectra were obtained using four-wavelength scanning with a resolution of 4 cm ^–1^ in the range 4000 to 400 cm^–1^.

Thermogravimetric analyses (TGA) of the prepared membranes were carried out in a TGA-DTA Shimadzu (Kyoto, Japan) under N_2_ flow at a pressure of 5 bar and a flow rate of 50 mL/min in the temperature range of 25–650 °C and a heating rate of 10 °C min^–1^, to assess the thermal stability of the MMMs, and elucidate the distinct decomposition processes at specific temperatures. Each membrane was measured twice using 1–5 mg of sample for each measurement.

### 2.5 Gas Separation Experiments

The prepared membranes were cut to an effective area of 15.55 cm^2^ and introduced into a stainless-steel module. The module consists of two pieces of stainless steel with a cavity in the middle, where the membrane is placed on a 316LSS microporous disk support with a pore size of 20 μm. The module is sealed by Viton rings and 8 screws.

**Figure 1:**
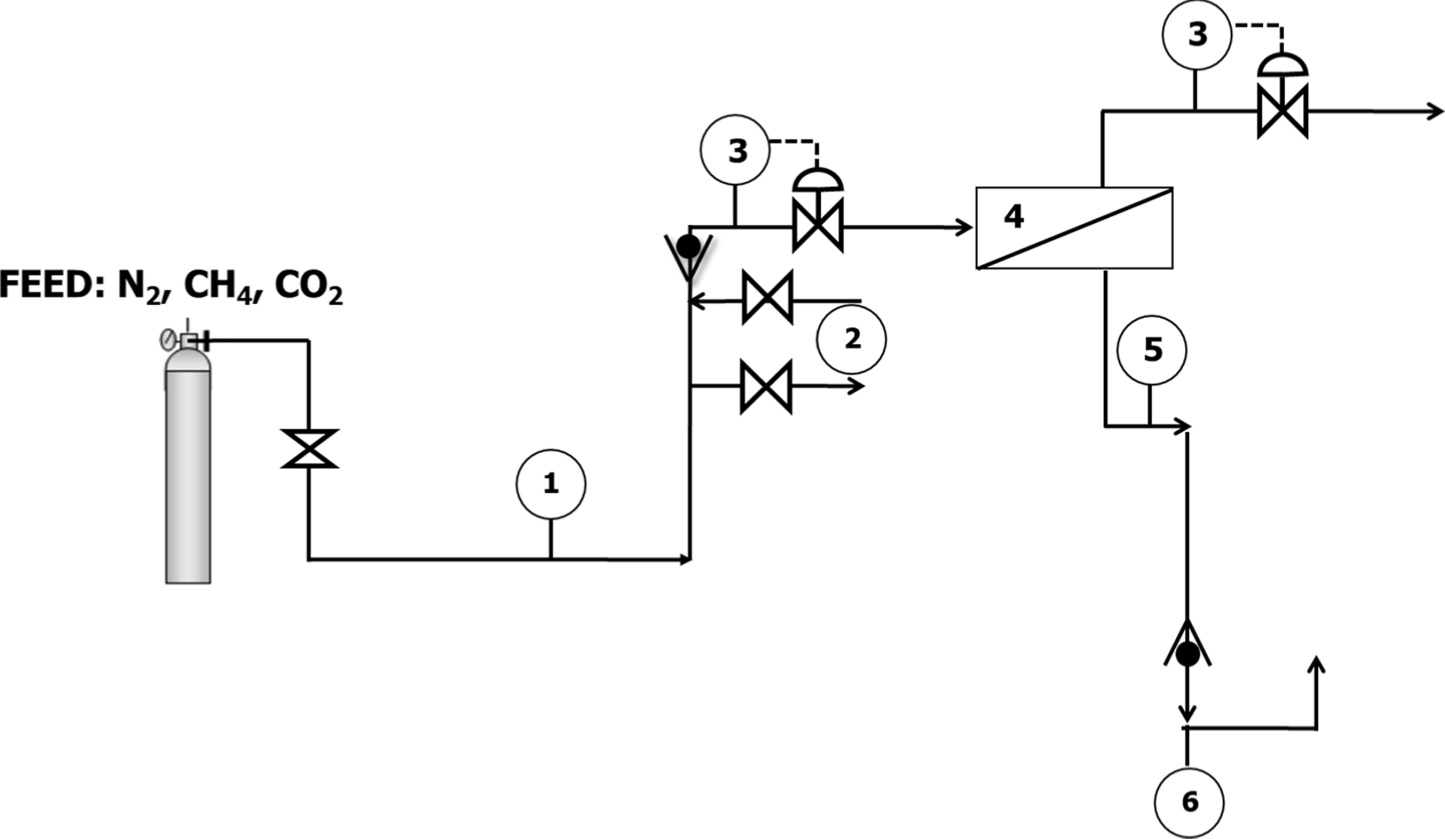
Experimental setup for the single-gas permeation experiments. (1) Mass flow controller; (2) water bubbler; (3) feed and retentate pressure regulator; (4) membrane modules; (5) permeate pressure indicator; (6) permeate flowmeter.

The experimental procedure used for the single gas permeation characterization measurements is described elsewhere.^51^ An experimental homemade bench-scale plant has been used for all the experiments (Figure 1). The feed pressure is set at 4 bar, and the experiments were run at room temperature, i.e., 20 ± 3.5 °C. Total feed flow rate was set in all the cases at 50 mL/min. The permeate flow rate was measured with a bubble flowmeter. The permeation measurements were taken once the system reached the steady state.

The eq 5 represents the gas permeability in terms of GPU (1 GPU = 10^–6^ cm^3^ (STP) cm^–2^ s^–1^ cmHg^–1^), defined as the flux of gas through a membrane normalized by the pressure.
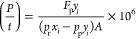
5where *P* is the intrinsic permeability of the desired gas across the membrane [Barrer, 1 Barrer = 10^–10^ cm^3^ (STP) cm cm^–2^ s^–1^ cmHg^–1^]; *t* is the thickness of the membrane (cm); *F*_p_ is the permeate flow rate [cm^3^ (STP) s^–1^]; *y*_*i*_ is the mole fraction of the component *i* in the permeate; *x*_*i*_ is the mole fraction of the component *i* in the feed; *p*_p_ and *p*_r_ are the permeate and retentate pressures (cmHg); *A* is the membrane area (cm^2^).

The selectivity is calculated as the ratio between the gas permeability pairs
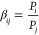
6

### 2.6 Models for the Prediction of the Gas Separation Performance

The experimental permeability data are validated with different theoretical expressions for the prediction of MMMs performance.^52^ The Maxwell model is one of the most commonly used models to the predict gas permeability of heterogeneous systems where particles are randomly dispersed in other continuous phases, as it provides a straightforward method for predicting the transport properties of a new hybrid membrane material when the permeability of the constituent phases is known.^53^ In this study, the Maxwell model was applied to validate the experimental behavior of the CA-DMC based MMMs, by eq 7, which is the most widely applied for predicting the permeability
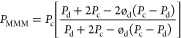
7where *P*_MMM_ is the predicted permeability of the membrane; ø_d_ is the volume fraction of a dispersed phase (d) in a continuous matrix phase (c); *P*_c_ and *P*_d_ are the permeabilities of the continuous and dispersed phases, respectively.^53^*P*_c_ is the experimentally measured permeability of the pure CA membranes prepared in this work. *P*_d_ is taken from literature or previous works, for pure zeolite NaA^54^ and ETS-10 membranes^55^ and ZIF-8.^56^ The volume fraction of the dispersed filler phase is calculated as
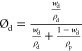
8where *w*_d_ is the weight fraction of the filler dispersed phase and ρ_p_ is the density of the continuous CA polymer, taken as 1.3 g/cm^3^ from the supplier. The density ρ_d_ was taken as the crystallographic density of the filler particles, for ETS-10 (1.75 g/cm^3^),^57^ AM-4 (2.74 g/cm^3^),^58^ and ZIF-8 (0.93 g/cm^3^),^56^ respectively.

The maximum limit of Maxwell equation model is referred, on the one hand, to parallel transport though a laminate, expressed by
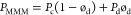
9

The minimum limit considers that the transport occurs though a laminate in series with the matrix, then eq 7 turns to
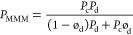
10

The Maxwell equation do not allow investigating the effect of size or shape of the nanoparticle fillers, which may be important in the final quality of the MMM. Therefore, other variations including the incorporation of different shapes have been reviewed.^59^ One of the most acknowledged equations account for the effect of layered (2D) versus (3D) fillers in a polymer matrix is the one derived by Nielsen, to predict the effect of flakes into a polymer matrix^60^ as
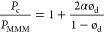
11where α is the aspect ratio of the width and thickness of a single particle estimating the filler shape. The aspect ratio of AM-4 was calculated as 24 from the dimensions of the particles measured in a previous work.^46^ The aspect ratio of ETS-10 was likewise calculated as 0.78.^49^ In this work, we compare Maxwell equations with Nielsen equations to observe the influence of particle morphology in the performance of CA-DMC MMMs.

## 3 Results and Discussion

### 3.1 Characterization Techniques

According to the HSP of CA in different solvents commonly used in CA membrane preparation (Table S2 in the Supporting Information), it is expected that CA exhibits good solubility in solvents with similar HSP values, especially those with moderate polar and dispersive characteristics (e.g., acetone, chloroform, dichloromethane), while it is insoluble in solvents with very different HSP (e.g., water). The interaction radius (*R*_a_), which is indicative of the polymer–solvent distance, where lower values indicate greater solubility, is higher for CA-DMC compared to the most common toxic solvents like DMF or DMAc, but similar to CA-acetone, with a value of 10.13. The solubility behavior of CA in these solvents will also depend on other factors such as molecular weight, degree of substitution, and temperature.^61^ As a result, the solubility of CA in DMC was confirmed, although the δ_p_ value is slightly lower than that of CA, while diethyl carbonate presents a δ_T_ that is far from the respective value of CA, which may point out the insolubility of CA in this solvent. The compatibility of CA and DMC solvent in the preparation of CA electro spun nanofibers was also observed by Oldal et al.^62^ The DMC was selected over greener solvents like dimethyl sulfoxide (DMSO) and triethyl phosphate (TEP), Polarclean, or Cyrene owing to its balance of environmental benefits, compatibility, and process efficiency.^40,63^ Its moderate boiling point and the possibility to produce totally dense membranes reinforced its suitability for gas separation applications in this study. In fact, the membrane produced with DMSO or TEP has been less frequently studied for gas separation but has shown potential for other filtration and separation applications such as ultrafiltration and nanofiltration. The reason is based on their high boiling point and viscosity, which can complicate processing, requiring precise control of temperature and drying conditions.

Table 3 collects the thickness, WU, and WCA, together with mechanical and thermal properties of the CA-DMC membranes. All the WCAs result in values smaller than 80°,^64^ which indicates that all the MMMs have a hydrophilic nature.^65^ The WCA of the pure CA-DMC membranes agree with the WCA of 63° reported for a commercial CA support.^66^ The 0.5 wt % ETS-10/CA-DMC MMM gives a WCA value of 56.3 ± 2.3°, highlighting the effect of ETS-10 titanosilicate nanoparticles in the biopolymer matrix as observed in a previous work on 5 wt % ETS-10/chitosan membranes.^45^ The WCA of ZIF-8 and ETS-10/CA-DMC show the same evolution with filler loading in this work, a minimum at the lowest 0.5 wt % filler loading, and then a stable value over 70°. The WCA of the Zeolite A/CA-DMC membranes is kept constant within the values of 62 and 67° for the whole filler loading range, in line with the hydrophilic nature of the zeolite. Different is the effect of layered titanosilicate AM-4 as filler, where the minimum value of WCA is shifted to 1.0 wt % instead of 0.5 wt %, due to the higher aspect ratio of this material.

**Table 3:** Thickness, WU, WCA, Mechanical Properties (Young Modulus, Elongation at Break) and Thermal Decomposition of the CA-DMC Membranes

membrane code	thickness [μm]	WU [%]	WCA [deg]	Young modulus, *E* (MPa)	elongation at break (%)	*T*_d_ (°C)
CA	27.0 ± 2.55	9.9 ± 0.2	66.0 ± 1.6	875 ± 102.6	15.8 ± 0	299.8 ± 0.4
0.5 wt % zeolite-4A/CA	34.0 ± 3.7	8.2 ± 3.0	71.8 ± 4.1	571 ± 62.9	18.5 ± 8.1	288.8 ± 9.9
1.0 wt % zeolite-4A/CA	27.5 ± 3.9	9.8 ± 5.0	70.8 ± 3.4	661 ± 98.3	20.1 ± 2.3	290.6 ± 2.8
2.5 wt % zeolite-4A/CA	20.4 ± 10.7	11.8 ± 1.8	67.1 ± 2.2	730 ± 114.1	17.8 ± 0	279.3 ± 2.2
0.5 wt % ETS-10/CA	15.8 ± 2.5	8.6 ± 0.9	56.3 ± 2.3	438 ± 101.0	29.0 ± 0.1	306.8 ± 3.7
1.0 wt % ETS-10/CA	23.3 ± 1.1	10.7 ± 2.5	75.2 ± 5.8	408 ± 88.0	23.4 ± 0	302.9 ± 1.7
2.5 wt % ETS-10A	22.5 ± 5.6	7.7 ± 1.4	74.0 ± 5.5	409 ± 17.7	20.7 ± 8.6	289.3 ± 4.6
0.5 wt % AM-4/CA	25.8 ± 3.8	9.2 ± 1.8	62.3 ± 1.3	388 ± 110.3	9.9 ± 5.3	273.3 ± 5.3
1.0 wt % AM-4/CA	23.0 ± 2.9	10.6 ± 0.1	51.6 ± 1.3	369 ± 2.1	14.6 ± 5.7	272.0 ± 3.5
2.5 wt % AM-4/CA	19.5 ± 2.6	29.3 ± 3.6	66.9 ± 4.3	350 ± 101.1	25.2 ± 6.8	257.0 ± 0.5
0.5 wt % ZIF-8/CA	25.4 ± 2.3	27.2 ± 4.4	56.6 ± 6.7	339 ± 124.8	9.3 ± 1.6	305.1 ± 2.8
1.0 wt % ZIF-8/CA	22.4 ± 3.1	28.4 ± 1.9	78.2 ± 4.8	185 ± 79.9	10.4 ± 6.4	286.6 ± 11.5
2.5 wt % ZIF-8/CA	22.3 ± 4.3	31.3 ± 4.6	66.0 ± 1.6	173 ± 23.3	14.5 ± 2.7	275.8 ± 2.1

**Figure 2:**
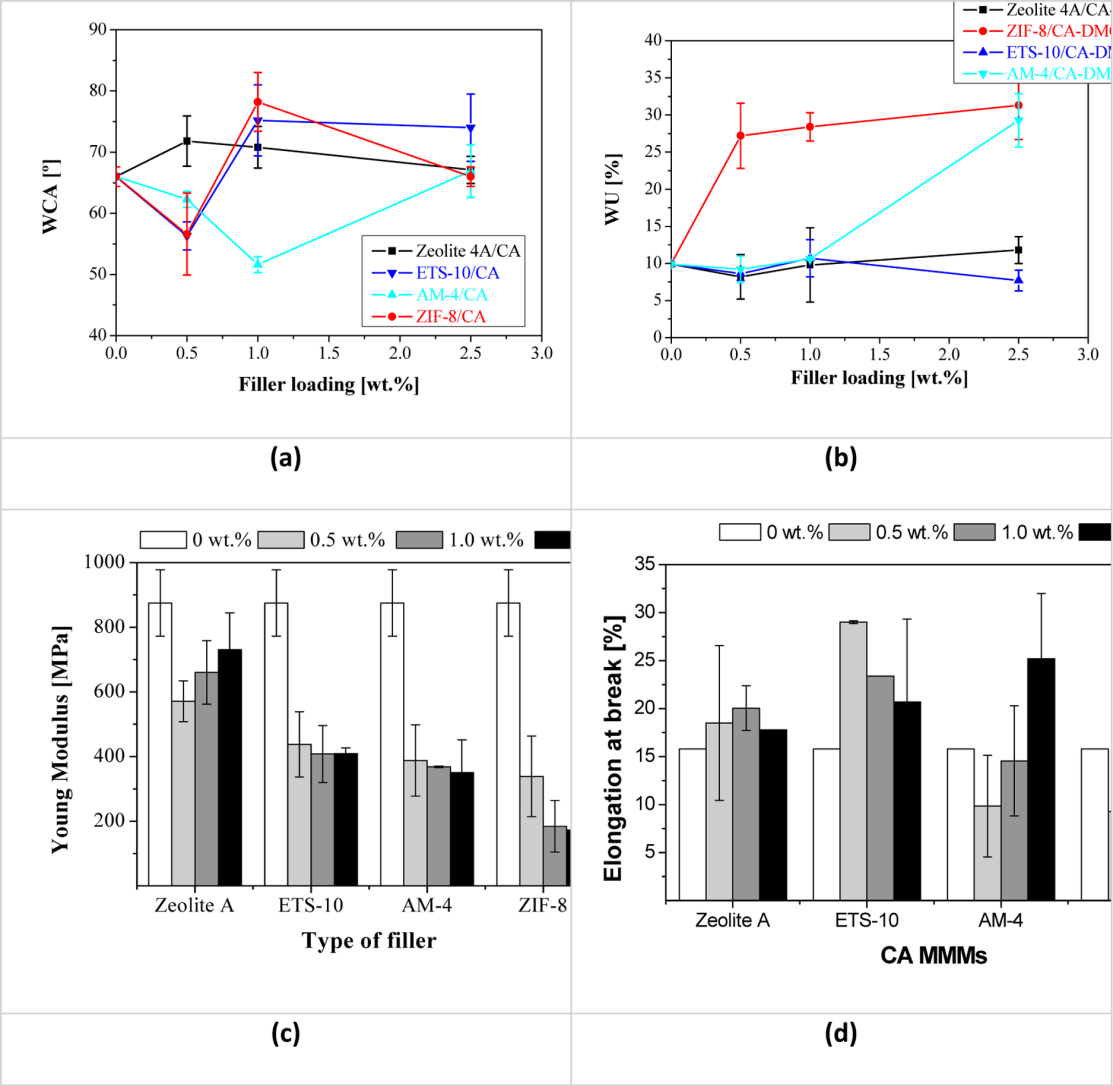
(a) WCA, (b) WU, (c) Young modulus, and (d) strain rate of the CA-DMC MMMs as a function of filler type and filler loading.

The WU property provides some indication of the moisture content in the membrane, with the highest hydrophilicity obtained for the ZIF-8/CA-DMC MMMs. This could be associated to the multidimensional structure fine pore distribution and the large surface area that this filler offers to the membrane, which provides sufficient storage capacity to adsorb water and CO_2_ molecules. In the other MMM studied in this work, this high WU values are only attained for the 2.5 wt % AM-4/CA-DMC, 30%, though in this case, this should be attributed to the larger aspect ratio and hydrophilicity of the lamellar AM-4 particles.^46^ Zeolite 4A and ETS-10 provided the lowest WU values in this work, despite the lower water contact angles that had been correlated to the high hydrophilicity of zeolite 4A and ETS-10 particles.^47,67^ Since the WCA technique provides information on the surface characteristics of the membrane, while WU values give insight on the core volume of the membrane, these results indicate that accessibility to the sorption sites of the porous fillers is more significant to the filler–polymer interaction than the hydrophilicity in the case of CA as compared with other biopolymers. Figure 2 represents the influence of the membrane filler type and loading content on the bulk (WU) and surface (WCA) moisture of the prepared membranes.

The mechanical properties of the prepared membranes are also collected in Table 3. The pristine CA membranes prepared in this work have a value in line with literature, in the range between those reported by Nazari et al.^64^ and Li et al.^29^ This can indicate a good affinity between the polymer and the solvent, as predicted by the HSP analysis. Kim et al. fabricated CA membranes using different green solvents with hardness up to 8 MPa and elongation at break up to 11%, but still need to be improved for high-pressure NF applications.^40^ However, the addition of fillers reduces significantly the value of the Young modulus of the MMM compared to the pure CA membrane.^29^ This phenomenon is revealing either a reduced adhesion between the CA macromolecules and the porous fillers, exacerbated by increasing loading, in agreement with the changes in hydrophilic character, i.e., WCA, as observed by Rehman et al.^68^ In fact, we observed that our CA-DMC membranes exhibited faster drying rates with increasing filler content, as evidenced by the WU and WCA values (Figure 2). This rapid drying may be compromising the mechanical structure integrity of the biopolymer membrane.

**Figure 3:**
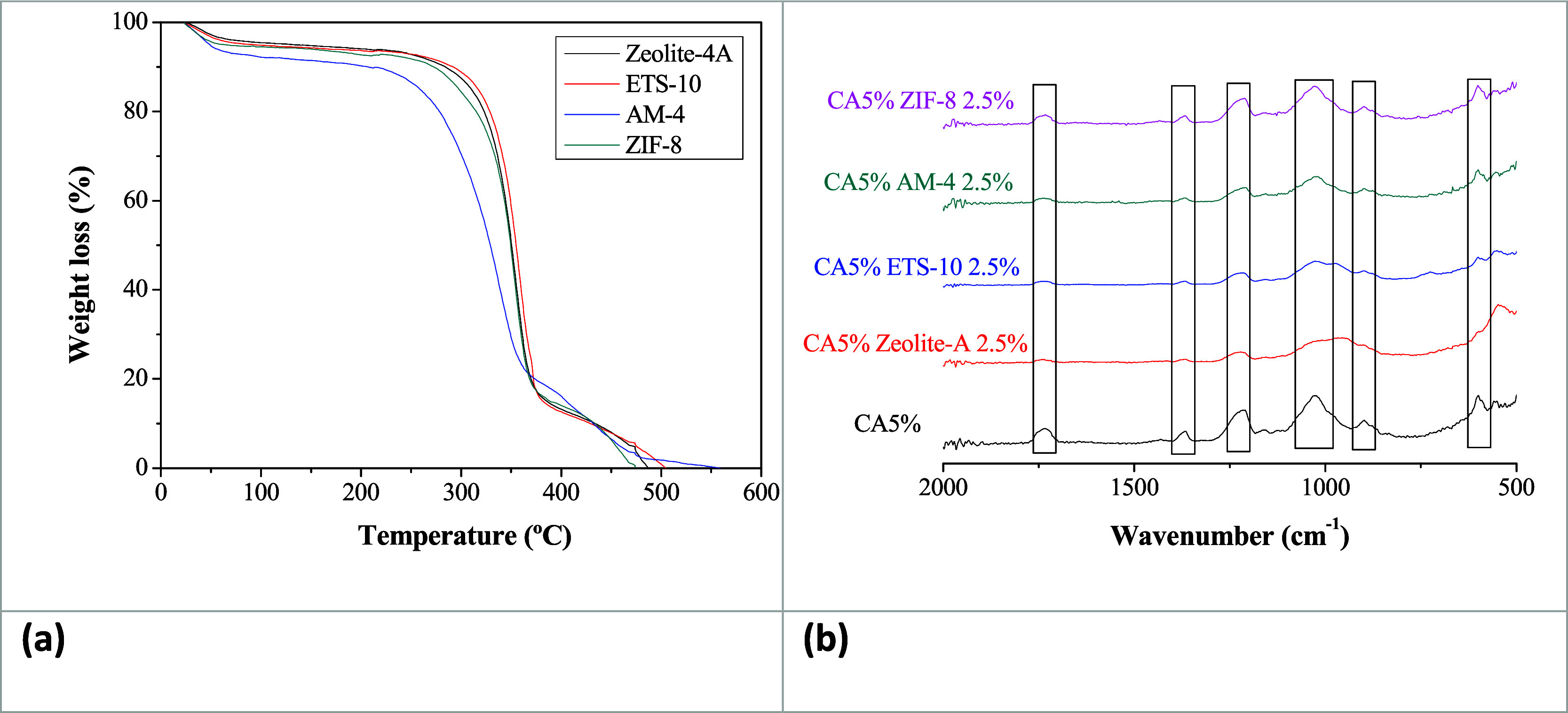
(a) TGA thermograms and (b) ATR spectra of the 2.5 wt % filled CA-DMC MMMs.

Regarding the thermal stability, a consistent pattern is observed for all the CA-DMC MMMs, where the onset decomposition temperature is slightly decreased with increasing filler loading.^29^ The thermograms for the 2.5 wt % filled CA-based MMMs are depicted in Figure 3a. The decomposition is characterized by three distinct stages, in agreement with the study by Abdullah et al., who prepared CA membranes using CA from different sources^65^ as well as the MgO/CA membranes reported by Rajpure et al.^23^ In the initial stage (50–100 °C), there is a weight loss of 10 wt %, attributed to the removal of excess water adsorbed on the membrane surface and the residual solvents. This agrees with the values obtained for the WU. The second stage (200–350 °C) accounts for the major decomposition of the polymer backbone, with a weight loss of 80% attributed to chain degradation of CA.^28,65^ The final decomposition process takes place at the temperature range of 350–525 °C that could be related to the carbonization of the decomposed polymeric chain.^23^ Regarding the effect of filler loading (not shown), zeolite A and ETS-10 at the low filler loading values used in this work do not provide any significant difference, but AM-4 and ZIF-8 do reveal differences in the first and last stages of the thermogram, associated with their different morphologies and nature. This agrees with other observations on ZIF-filled CA MMMs showing different char residues at the end of thermal degradation with increasing ZIF loading.^28^

The chemical composition of the CA-DMC MMMs was examined by ATR-FTIR after permeation measurements. Figure 3b only shows the spectra of the 2.5 wt % filled membranes in the range from 500 to 2000 cm^–1^, in order to distinguish the intrinsic bands of the respective fillers. The CA membrane exhibits a distinctive band at 600 cm^–1^, due to the C–H group.^30^ The band at 900 cm^–1^ is associated with the acetate methyl group present in the polymeric structure.^69,70^ The asymmetric and symmetric ester C–O–C stretching modes appear as strong bands at 1215 and 1030 cm^–1^, respectively.^23,69^ Furthermore, a distinct peak at 1362 cm^–1^ indicates CH_2_ bending vibrations specific to CA, in accordance with the study carried out by Khamwichit et al.^35^ Additionally, a peak is discernible at 1730 cm^–1^ indicating the presence of the carbonyl group (C=O) within the CA matrix.^71^ The intensity of the peaks in the ATR-FTIR spectra of all the prepared CA-DMC MMMs decreases upon the incorporation of fillers, as observed by Tanvidkar et al.,^26^ confirming the successful integration of the particles into the CA matrix. A comprehensive analysis of the ATR-FTIR spectra not only confirms the successful synthesis of CA MMMs but also delineates the specific functional groups and their corresponding vibrations, contributing to a deeper understanding of the chemical composition of the membranes.

**Figure 4:**
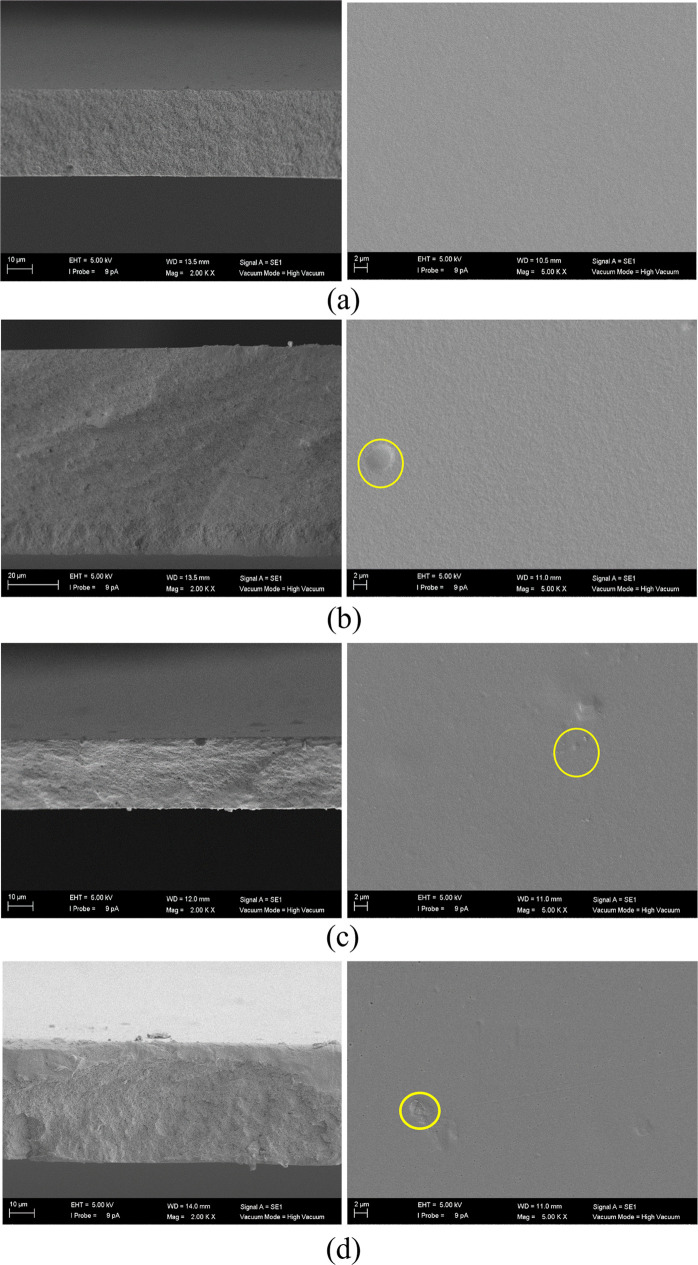
SEM images of the (a) 0, (b) 0.5, (c) 1.0, (d) 2.5 wt % zeolite A/CA MMMs. Left-hand pictures correspond to cross section (magnification ×2000, from top to bottom, respectively) and right-hand pictures to the top surface (magnification ×5000, from top to bottom, respectively) view of the membranes.

**Figure 5:**
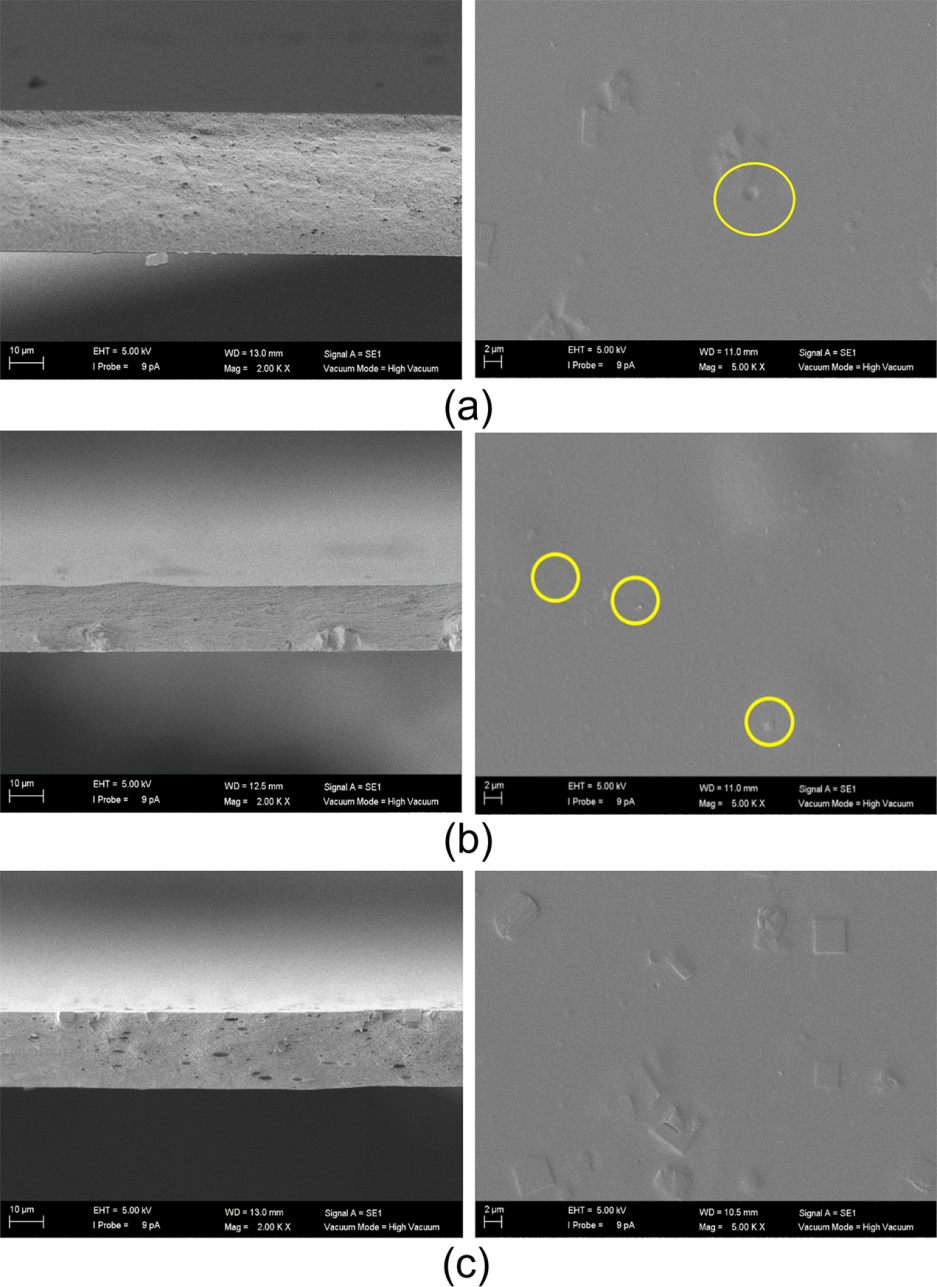
SEM images of the (a) 0.5, (b) 1.0, and (c) 2.5 wt % ETS-10/CA MMMs. Left-hand pictures correspond to the cross section (magnification ×2000) and right-hand pictures to the top surface view (magnification ×5000, from top to bottom, respectively) of the membranes.

**Figure 6:**
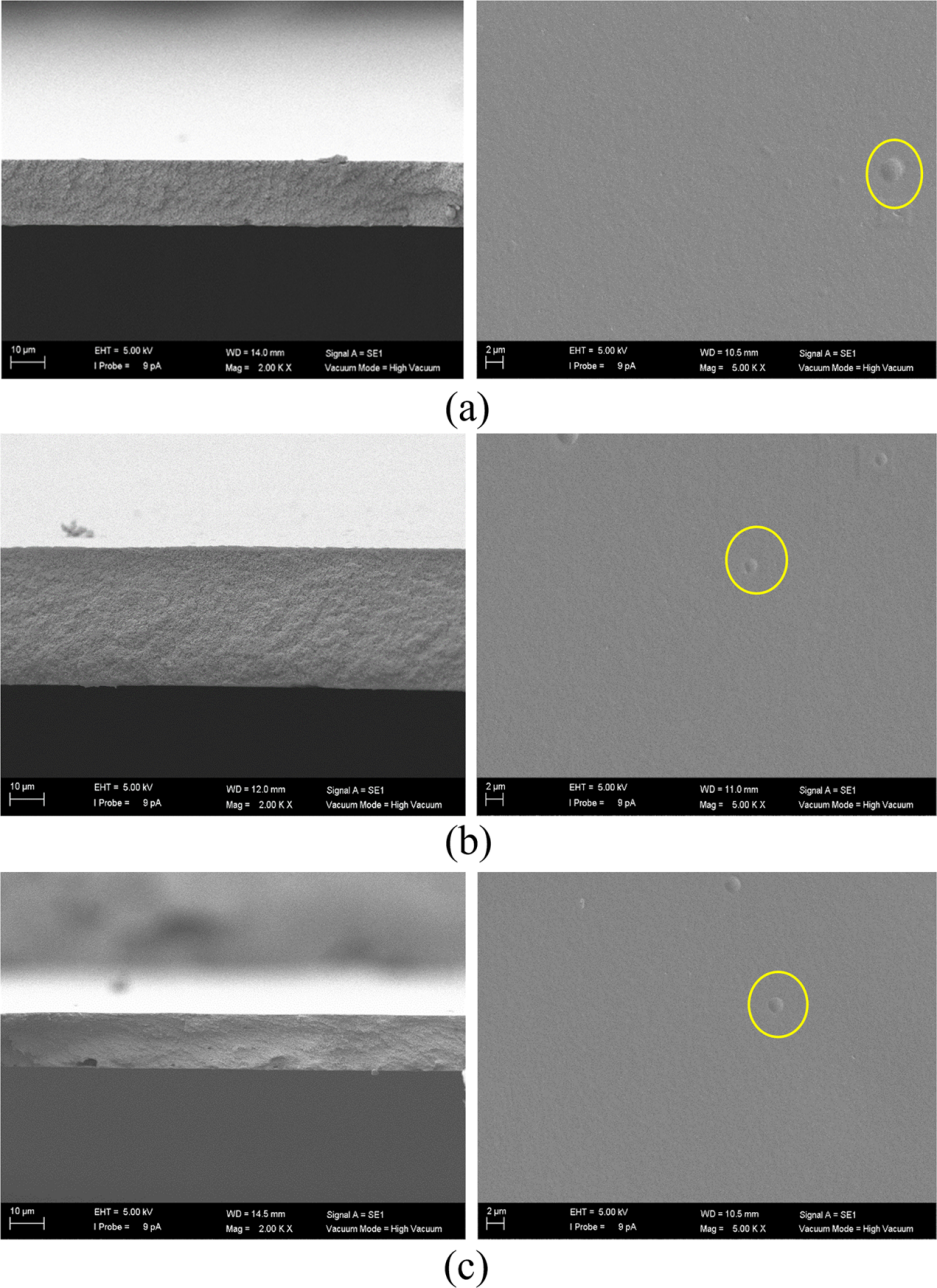
SEM images of the (a) 0.5, (b) 1.0, and (c) 2.5 wt % AM-4/CA MMMs. Left-hand pictures correspond to the cross section (magnification ×2000, from top to bottom, respectively) and right-hand pictures to the top surface view (magnification ×5000) of the membranes.

**Figure 7:**
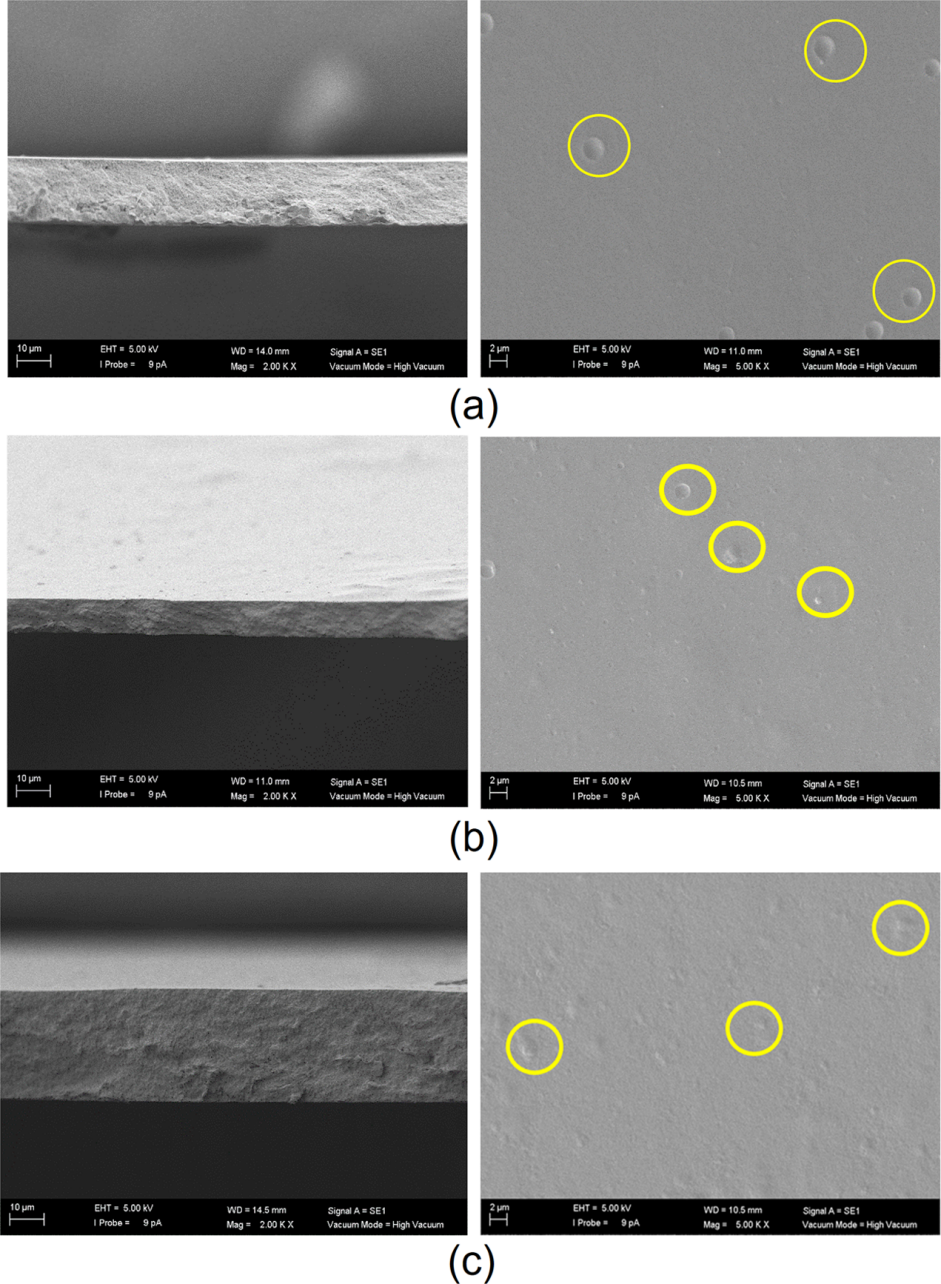
SEM images of the (a) 0.5, (b) 1.0, and (c) 2.5 wt % ZIF-8/CA MMMs. Left-hand pictures correspond to the cross section (magnification ×2000, from top to bottom, respectively) and right-hand pictures to the top surface (magnification ×5000, from top to bottom, respectively) view of the membranes.

The SEM images of the cross-section (left) and top surface (right) of the membranes are presented in Figures 4–7. Examining Figure 4a with respect to the pristine CA membrane, the top surface exhibits uniformity, smoothness, density, and lack of defects, indicative of a favorable polymer–solvent interaction. This observation aligns with findings in existing literature.^30,31^ The cross-sectional view of the CA agrees with the thickness measured by the Mitutoyo micrometer.

On the other hand, the top surface of the MMMs facilitates the observation of occasional agglomerations upon increasing filler loading, but no apparent phase separation is evident, underscoring the compatibility between the fillers and CA. It is crucial to look at Figure 5c, where the distinctive shape of the 3D titanosilicate ETS-10 is clearly depicted within the polymer matrix at the highest loading of 2.5 wt %, confirming the presence and effective interaction of this filler within the polymer, with a density close to the CA, enabling the particles to approach the surface (as highlighted in the enclosed circles, in yellow in the web version). However, the cross-sectional views in Figure 5 reveal the presence of voids.

### 3.2 Gas Permeation Experiments

**Figure 8:**
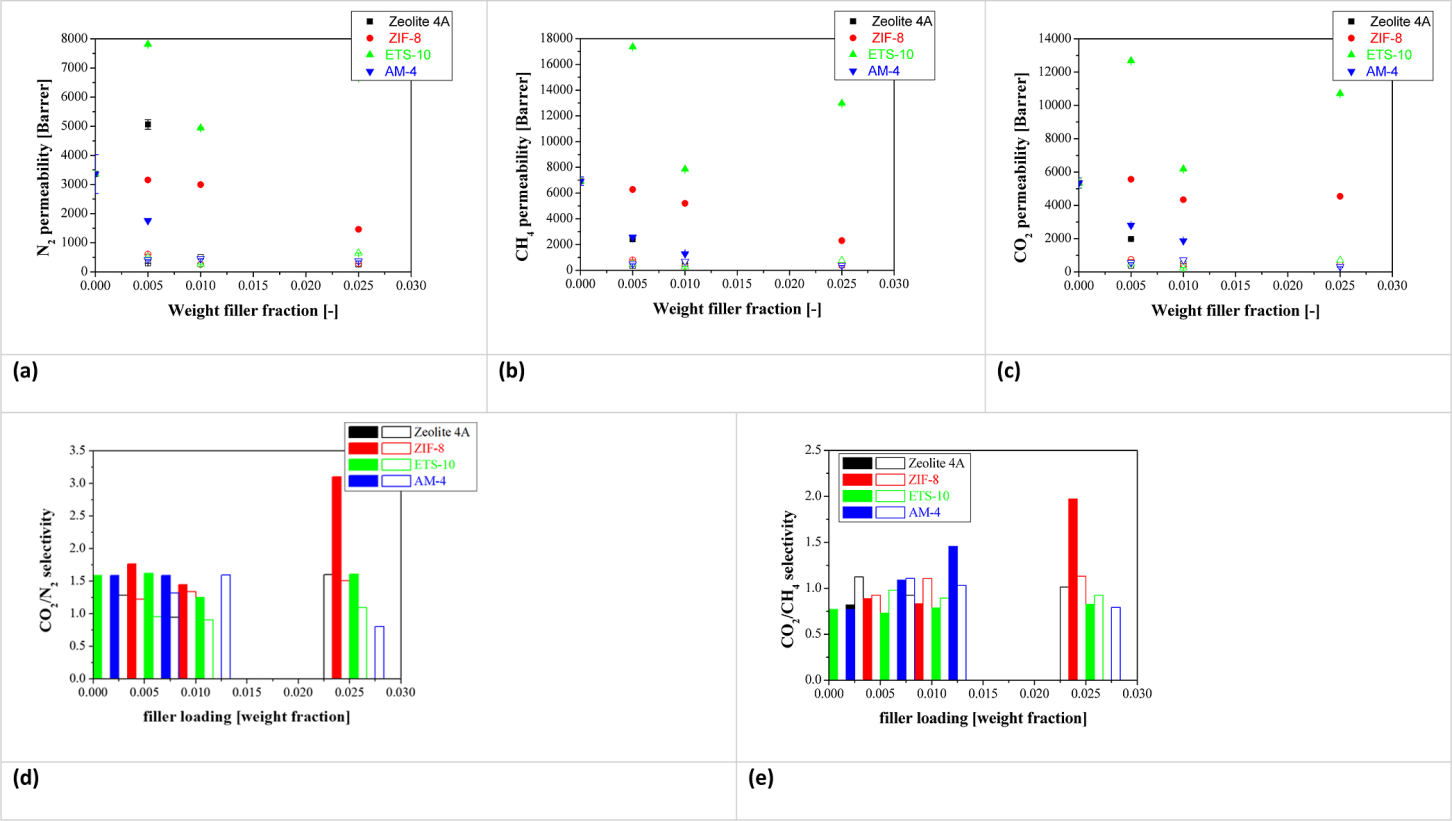
Single gas permeability values of CO_2_ (a), CH_4_ (b), and N_2_ (c) of the CA-DMC membranes as a function of filler loading. Ideal selectivities for the CO_2_/N_2_ (d) and CO_2_/CH_4_ (e) gas pair mixtures. Full and void symbols and bars correspond to the data obtained with the fresh (full) and conditioned (void) membranes, respectively.

The gas separation performance of the prepared membranes was studied by measuring the single gas permeabilities at 4 bar and ambient temperature of N_2_, CH_4_, and CO_2_, in that order, to prevent undesired CO_2_-induced plasticization effects on the characterization of the transport properties of the membrane. They are represented as a function of filler loading in Figure 8a–c. From these results, we distinguish two distinct orders of magnitude of permeability values: at the beginning of the permeation experiment, the permeability was generally above 10,000 Barrer and after 1 h, the permeability dropped 10 to 100-fold. Mubashir et al.^30^ also observed a decrease in CO_2_ permeability from pressures of 3 bar attributed to the dual sorption characteristic of CA, which reduced the CO_2_ solubility coefficient, and was associated with the plasticization of CA and reduced the CO_2_ solubility coefficient. This decrease in permeability at a pressure of 3 bar confirms that some kind of conditioning^72^ is occurring but neither plasticization nor swelling of the glassy polymer matrix is expected at the operating pressure of this work.^73^

Because of this conditioning, there is a decrease in CO_2_/N_2_ and CO_2_/CH_4_ selectivities plotted in Figure 8d,e. This has been attributed to the saturation and limited flexibility of the fillers, as well as the existence of nanodefects at the polymer–filler interface, being able to absorb both gas molecules when the kinetic diameter of the gas molecules to be separated is very close.^28^ This explains why the CO_2_/CH_4_ selectivity of ZIF-8/CA-DMC fresh MMMs doubles that obtained at lower loadings by the breathable nature of the ZIF-8. The low selectivity values are nevertheless in line with other CA-based membrane characterization reports found in literature. For instance, with TiO_2_/CA membranes reported CO_2_/CH_4_ selectivities below 0.5 due to the fact that CH_4_ molecules can easily pass through the chain volumes of TiO_2_/CA due to the nonexistence of tortuous paths.^22^ Farrukh et al.^74^ introduced TiO_2_ NPs in CA using acetone and dioxane as solvents and obtained a selectivity of 1.43, with CO_2_ permeabilities around 1000 Barrer. The novelty of our work is the exploration of the use of a different green solvent for CA in the preparation of gas separation membranes.

The resultant conditioned membranes seem to have attained the steady-state^72^ and thus the following discussion will focus on these values. In this state, the incorporation of porous fillers increases the CO_2_ permeability, from 314 Barrer (pure CA membrane) to 484 Barrer for the 1 wt % zeolite 4A/CA-DMC, 707 Barrer for the 2.5 wt % ETS-10/CA-DMC, 719.5 Barrer for the 1 wt % AM-4/CA-DMC, and 740 Barrer for the 0.5 wt % ZIF-8/CA MMM. The increased CO_2_ permeability can be explained because the porous fillers provide a faster pathway for gas transport,^27^ and this is achieved at lower loading in the ETS-10 and ZIF-8 filled MMMs. However, the permeability of ZIF-8/CA-DMC MMM is reduced when increasing ZIF-8 loading to 2.5 wt %.

Usually, the CO_2_ permeation mechanism occurs by surface diffusion, adsorption, and diffusion through the polymeric matrix, while the N_2_ permeation mechanism is only diffusion and adsorption, which justifies the higher CO_2_ permeability than N_2_ permeability.^75^ In this work, the highest CO_2_/N_2_ selectivity is attained by the 2.5 wt % ZIF-8/CA MMM, and this is attributed to the better affinity of this MOF with CA.^27^ Jin et al.^76^ prepared membranes by combining two types of CA at different acetyl concentrations, 39% and 56%, and obtained a CO_2_/N_2_ selectivity of 1.14, which was attributed to the small thickness of the selective layer (Table 3), implying a lower resistance against the passing gas and thus causing lower selectivity. Farrukh et al.^77^ reported selectivity values close to 1, for CA membranes filled with a calixarene derivative in different combinations of both materials, which were finally related to a specific host–guest interaction between the calixarene derivative and the CO_2_ molecule. The nascent trend toward particle agglomeration of particles near the membrane surface observed by SEM (Figures 4–7) may also be a reason for the low selectivity and the increasing permeability.^78^ Abdelgadir et al.^79^ correlated the CO_2_ permeabilities up to 4850 Barrer with CO_2_/N_2_ selectivities of 0.8 in their MWCNT/CA MMMs to the agglomeration of the fillers in the membrane surface with increasing loading.

### 3.3 Comparison of Gas Permeation Results with Literature Data

**Figure 9:**
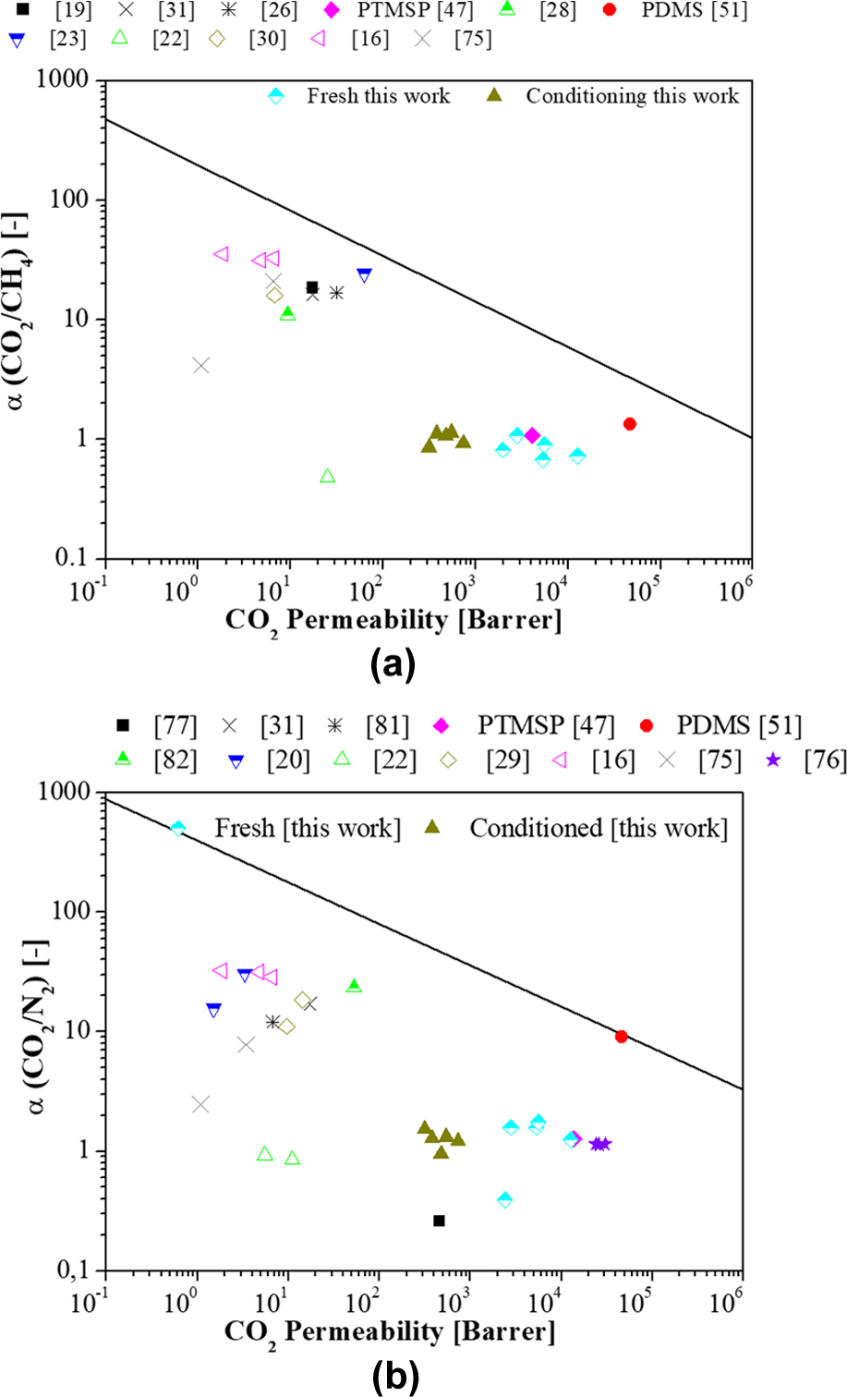
Robeson’s upper bound for the CO_2_/CH_4_ (a) and CO_2_/N_2_ (b) permeability-selectivity trade-off.^81,82^

The separation performance of the MMMs prepared in this work is compared with previously reported data of CA-based membranes using the Robeson upper bound for CO_2_ binary mixtures.^80^Figure 9 represents the trade-off between CO_2_ permeability and CO_2_/CH_4_ selectivity (a) and CO_2_/N_2_ selectivity (b), respectively. Most data on CA membranes in the literature report higher selectivity and lower permeability values than the CA-DMC based membranes in this work for CO_2_/CH_4_ separation. The highest CO_2_/N_2_ selectivity, found in literature, was achieved by the combination of CA acetylation degrees (2.45% and 2.84%) in methylene chloride and acetone as solvents, since the seminal work of Puleo et al.^16^ Jin et al.^76^ combined CA with two acetyl contents of 56% and 39%, using acetic acid as solvent, and obtained CO_2_/N_2_ selectivities of 1.14 observing no influence in the acetylation degree of CA. On MMM approach to improve CA membranes, Rashid et al.^22^ tried to improve the CO_2_/N_2_ separation performance of CA membranes by incorporating 2 wt % TiO_2_ using THF as solvent, thereby increasing the CO_2_ permeability from 5.5 to 11 Barrer but decreasing the CO_2_/N_2_ selectivity from 0.95 to 0.85.

**Figure 10:**
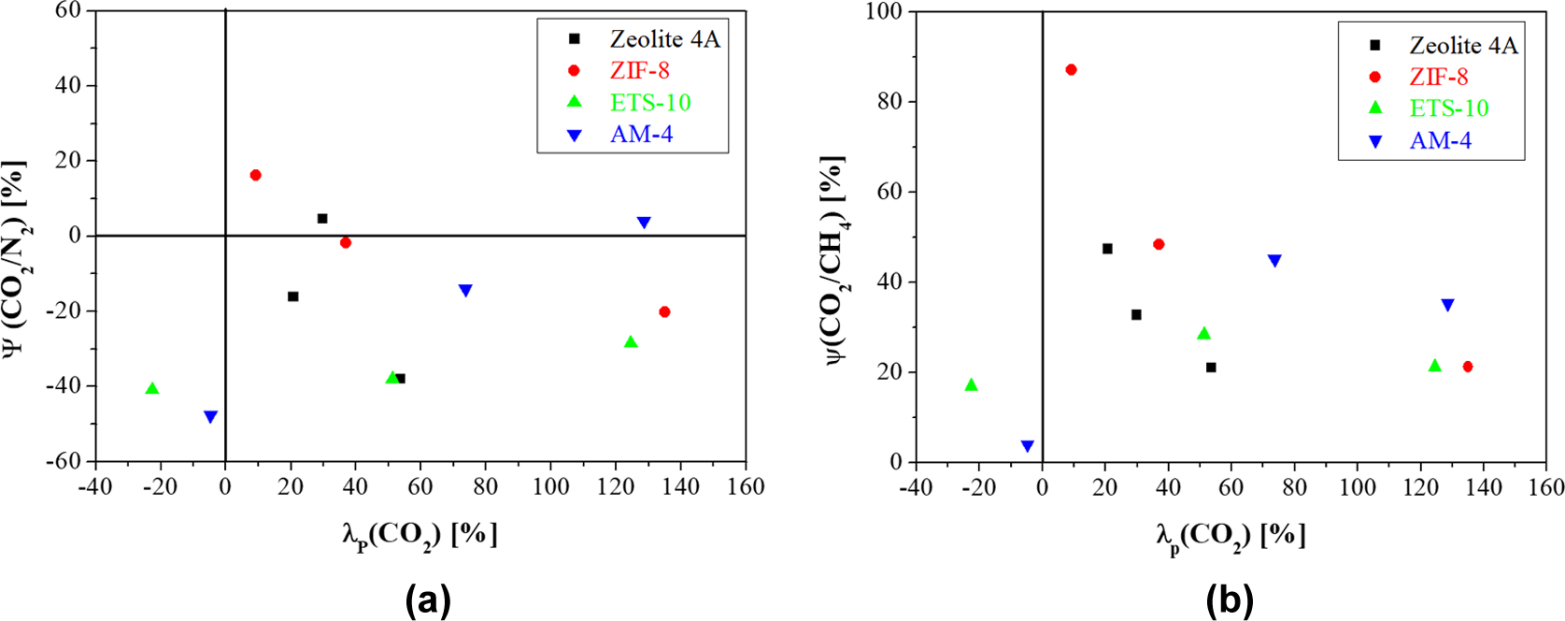
Selectivity enhancement of CA-DMC based MMMs vs CO_2_ permeability enhancement for (a) CO_2_/N_2_ and (b) CO_2_/CH_4_ separation, respectively.

In order to make renewable and nontoxic CA-based membranes using DMC solvent, the chemistry interactions between CA, DMC, and compatible fillers have to be evaluated. Since the Robeson diagrams cannot separate the intrinsic effect of the continuous polymer matrix from the true effect of the filler and the synthesis method, a more comprehensive understanding of the CO_2_ separation evaluation of the CA-DMC MMMs, the permeability and selectivity enhancement diagrams developed by Pazani et al.^83^ and Maleh and Raisi et al.^84^ are applied below. The parameters developed by Pazani et al.^83^ are thus calculated for CO_2_/N_2_ and CO_2_/CH_4_ separation in Figure 10, as
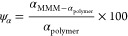
12
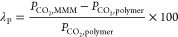
13

The enhancement factor in eq 12 reveals no actual enhancement regarding the performance of CA-DMC based membranes in CO_2_/N_2_ separation, but for CO_2_/CH_4_ separation, better insight might be achieved into the relative influence of the synthesis of CA MMMs using the *F*-index value, defined as
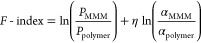
14

**Figure 11:**
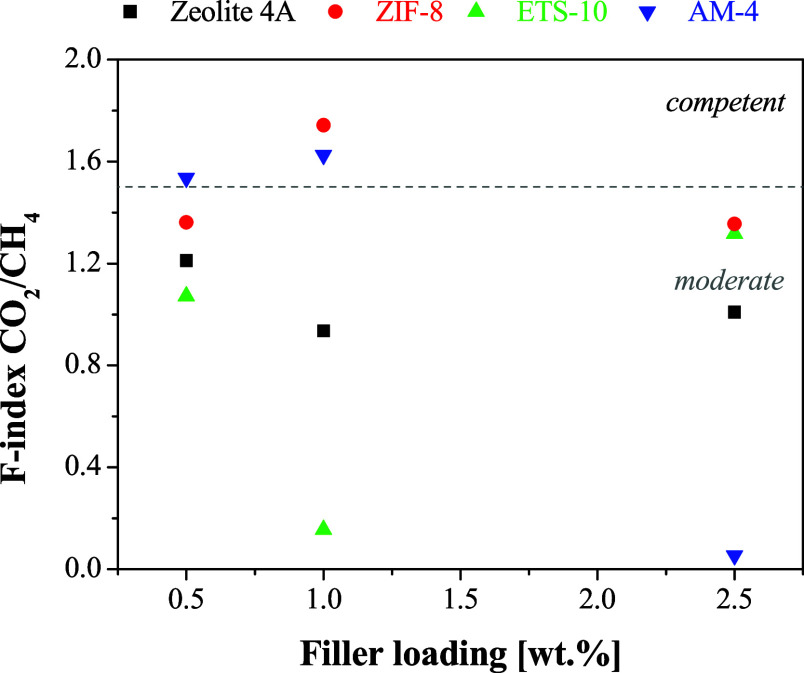
*F*-index of the prepared CA-DMC MMMs for CO_2_/CH_4_ separation.

The enhancement coefficient (η) approaches the slopes (*n*) of the 2008-upperbound.^83^ The *F*-index expresses the gas separation quality of MMMs in the ranges <0, 0–1.5, 1.5–4, 4–8, and >8, representing insufficient, moderate, competent, exemplary, and ideal qualities, respectively.^83^ As plotted in Figure 11, the CA-DMC MMM prepared in this work have moderate to competent performance, as a function of filler type and compatibility, which will be discussed below.

### 3.4 Analysis of the Experimental Results with Transport Theoretical Models

Maxwell derived models often represent the ideal case with no defects and no distortion of separation properties by the interaction of the filler and the polymer matrix. Table 4 summarizes the relative error (AARE) values obtained using Maxwell eq 7 and their minimum and maximum derivatives in eqs 9 and 10, series and parallel, respectively, for the membranes that showed a predictive error lower than 25%.

**Table 4:** Percentage of the Average Absolute Relative Error (AARE) for CO_2_, CH_4_, and N_2_ Permeability Prediction, Highlighting Those AARE Values Lower Than 20%

MMM	Maxwell			parallel			series		
	CO_2_	CH_4_	N_2_	CO_2_	CH_4_	N_2_	CO_2_	CH_4_	N_2_
0.5 wt % zeolite 4A	**22.2**	**15.9**		**22.2**	**16.7**	46	23	≫100	≫100
2.5 wt % zeolite 4A	36	**9.2**		35	5.4	33	41	≫100	≫100
1.0 wt % ZIF-8	**6.8**	**21.2**	**23**	**0.78**	**25**	**20**	**8.2**	**25**	**24**
2.5 wt % ZIF-8	30	**2.2**	33	**14.9**	9.2	27	34	**8.6**	36
0.5 wt % ETS-10	51	**18**	144	51	**18**	144	51	**22**	147
1.0 wt % ETS-10	**23**	33	31	**23**	33	32	**23**	30	34
0.5 wt % AM-4	51	**18**	**18**	99	51	**18**	51	**19**	145
1.0 wt % AM-4	123	87	216	98	126	86	**23**	31	32

According to the results, N_2_ could not be predicted by the Maxwell equations with acceptable error for the MMMs under study. The prediction accuracy of the CO_2_ and CH_4_ permeabilities varies as a function of the compatibility of the filler, as analyzed earlier in this work. This compatibility is influenced by the hydrophilic character of the particles compared with the MMM, the organic nature, since the ZIF-8 gives generally the best prediction, and the shape of the particles. No good fit was obtained at the highest loading studied in this work with the lamellar AM-4 titanosilicate.

Since no voids or defects due to poor interaction between polymer and fillers were detected upon SEM observation above (Figures 4–7), the nature of these deviations may be due to the blockage of the sieve pores by residual solvent or adsorbed polymer or the reduction of the mobility of the polymer chain near the sieve surface.^85^ Generally, the Maxwell equation underestimates the CO_2_ permeability as observed repeatedly in the literature. Chaidou et al.^86^ attributed these discrepancies on their Zeolite-4A/Matrimid 5218 MMMs to the poor contact between the polymer chains and the zeolite surface, forming interface voids that allows bypass around the sieve. Hu et al.^87^ observed that different ZIF-8 loadings added to PEGMEA and PEGDA led to Maxwell predictions below the experimentally obtained data. In this work, the underestimation of the model equations of the experimental CO_2_ permeability depends on the type of filler used, rather than the filler content,^86^ because the addition of different types of nanoparticle fillers alters the CA-DMC polymer–solvent structure in different ways.

**Figure 12:**
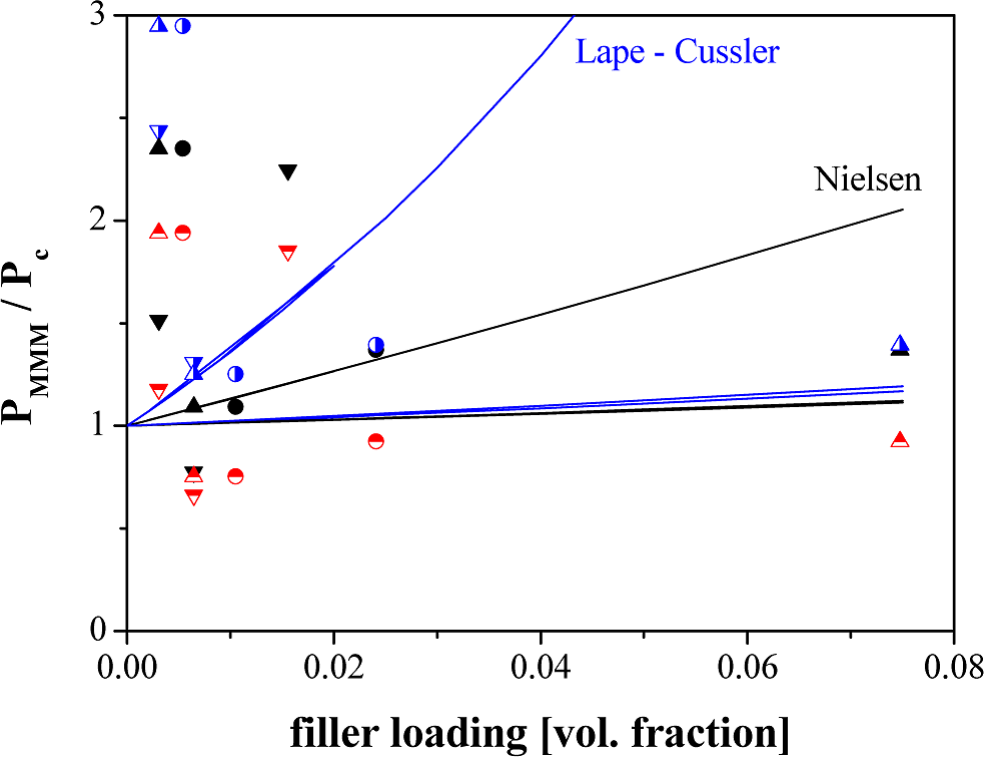
Theoretical curves generated from Nielsen and Lape–Cussler equations for the CA-DMC MMMs.

Some of the highest deviations are observed for AM-4-filled membranes at high loadings and for the 3D porous inorganic fillers at low loadings. According to the Nielsen equation, the deviations between experimental and theoretical values were the highest at low filler loadings. Nielsen model eq 11 has been applied to assess the effect of the aspect ratio on the permeation performance. Figure 12 shows the theoretical curves generated from Nielsen model versus the experimental data points of the CA-DMC with the fillers with different aspect ratio (ZIF-8, Zeolite 4A, α = 1; ETS-10, α = 0,78, and AM-4, α = 24). Nielsen model was first developed for ribbon like flakes, and AM-4 introduces nanoporosity which can lead to nonidealities difficult to explain yet, as Macher et al.^59^ discussed in their review. These nonidealities are also related to the fact that the model estimates a single flake, implying a complete exfoliation of the layers of the AM-4 particle by the dispersion into a compatible polymer matrix. When it is not the case, the aspect ratio varies from the value of 24 calculated from the average dimensions of a single layer (24, for the AM-4 structure), revealing that the layers are only partially delaminated.^60^ Thus, we included in Figure 12, the calculations using the Lape–Cussler model prediction are also added for comparison (eq 15), since it accounts for the lamellar flakes’ polydispersity in a random array and not perfectly aligned in the polymer matrix. In fact, a better approximation for both titanosilicate filled CA-DMC MMMs is observed, thus revealing the impact of the shape into the permeation performance.
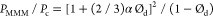
15

This is attributed to the hierarchical structure of the ETS-10, and the high hydrophilicity that may hinder the interaction with glassy CA, among others, making necessary the application of more complex modes to account for nonidealities as rigidification of the matrix, irregular interaction in the interface between the filler and the polymer, as investigated before.^45^

## 4 Conclusions

This work reports the fabrication of membranes of CA using for the first time the green solvent DMC. MMM (MMMs) of this polymer–solvent system were also prepared using different types of porous fillers prepared without costly organic surfactants or critical or toxic reactants, such as zeolite 4A, ETS-10 3D titanosilicate AM-4 2D titanosilicate, and ZIF-8 nanoparticles, at loadings of 0.5, 1, and 2.5 wt %. The introduction of fillers with hydrophilic character increased the WU in the bulk while decreasing the contact angle, as observed for ETS-10. The homogeneous dispersion and thickness of the membranes are generally corroborated by SEM. ATR-FTIR revealed the characteristic peaks of CA, confirming the successful synthesis of CA membranes and the interaction between CA and porous fillers. Pure gas permeability results indicated a conditioning step where the permeability of the membranes decreased in 100-fold for all gases, but no further increase was observed; so, no CO_2_ plasticization was expected. The CO_2_ permeabilities of the conditioned membranes were improved 35%, 57%, 53%, and 42% for Zeolite 4A, ZIF-8, ETS-10, and AM-4/CA-DMC MMMs, at 0.5 wt %. Only ZIF-8 -filled CA-DMC MMMs gave selectivity values higher than unity. The selectivity values are low but align with those of other novel blends of CA based membranes reported in the literature, showing potential for enhancement in a moderate range. The validation of these data with phenomenological model equations confirms the hypotheses that the shape and geometry of the fillers also play a role in the structure–performance relationship that should be explored in future works.

Although the membranes prepared in this work are still below the performance of commercial fossil-based gas separation membranes for biogas upgrading, the combination of an abundant biopolymer as a polymer matrix with DMC, as a low-toxicity green solvent and compatible porous fillers prepared without toxic solvents, will certainly offer new crucial insights into the sustainability of gas separation membranes in the future.

Future research should focus on advanced surface modification techniques for fillers or the development of innovative compatible agents to reduce interfacial defects and enhance filler dispersion, thereby improving both the selectivity and permeability. Additionally, exploring alternative filler types or combinations with diverse morphologies and pore architectures could address performance limitations and optimize gas transport pathways. Furthermore, refining membrane synthesis parameters, such as solvent evaporation rates, polymer concentration, and drying conditions, may help minimize structural nonidealities and the absolute average relative error (AARE %). These efforts will pave the way for the development of high-performance, sustainable MMMs membranes, advancing their application in gas separation.

## Supporting Information Available

Synthesis and characterization of CA membranes for different applications and the calculated HSPs of different fossil-fuel-based solvents used in the industry and some green solvents compared to CA (PDF)
