# Supplementary material for: Green Synthesis of Cellulose Acetate Mixed Matrix Membranes: Structure–Function Characterization
Source: ACS Sustain Chem Eng. 2025 Jan 16;13(3):1253–70. doi: 10.1021/acssuschemeng.4c07538 (PMC11776891; doi:10.1021/acssuschemeng.4c07538)
Supplement: Supplementary file 1 — sc4c07538_si_001.pdf [file sc4c07538_si_001.pdf]

Green synthesis of cellulose acetate mixed matrix membranes: Structure-function characterization

Andrea Torre-Celeizabal[1], Francesca Russo[2], Francesco Galiano[2], Alberto Figoli[2], Clara Casado-Coterillo[1], Aurora Garea[1]

1 Department of Chemical and Biomolecular Engineering, Universidad de Cantabria, Av. Los Castros s/n, 39005 Santander, Spain

2 Institute on Membrane Technology (CNR-ITM), Via P. Bucci 17/C, 87036 Rende (CS), Italy

Supplementary Information

Summary of tables in the manuscript and supporting information

Table 1. Review of literature on CA-based membranes for gas separation.

Table 2. List of the CA-DMC membranes prepared and characterized in this work.

Table 3. Thickness, water uptake (WU), water contact angle, mechanical properties (Young modulus, elongation at break) and thermal decomposition of the CA-DMC membranes.

Table 4. Percentage of the average absolute relative error (AARE) for CO_2, CH_4 and N2 permeability prediction, highlighting those AARE values lower than 20%.

Table S1. Relevant literature found on the synthesis and characterization of CA membranes for different applications.

Table S2. Hansen Solubility Parameters (HSPs) of different fossil-fuel-based solvents used in the industry and some green solvents compared to cellulose acetate (CA).

Summary of figures in the manuscript and supporting information

Figure 1. Experimental set up for the single-gas permeation experiments. 1 Mass flow controller; 2 water bubbler; 3 feed and retentate pressure regulator; 4 membrane modules; 5 permeate pressure indicator; 6 permeate flowmeter.

Figure 2. (a) Water contact angle, **(b)** Water Uptake**, (c)** Young modulus and **(d)** strain rate of the CA-DMC MMMs as a function of filler type and filler loading.

Figure 3. (a) TGA thermograms and **(b)** ATR-FTIR spectra of the 2.5 wt.% filled CA-DMC MMMs.

Figure 4. SEM images of **(a)** 0, **(b)** 0.5, **(c)** 1.0**, (d)** 2.5 wt.% Zeolite A/CA MMMs. Left-hand pictures correspond to cross section (magnification x2000, from top to bottom,

respectively) and right-hand pictures to the top surface (magnification x5000, from top to bottom, respectively) view of the membranes.

Figure 5. SEM images of **(a)** 0.5, **(b)** 1.0, **(c)** 2.5 wt.% ETS-10/CA MMMs. Left-hand pictures correspond to cross section (magnification x2000) and right-hand pictures to the top surface view (magnification x5000, from top to bottom, respectively) of the membranes.

Figure 6. SEM images of **(a)** 0.5, **(b)** 1.0, **(c)** 2.5 wt.% AM-4/CA MMMs. Left-hand pictures correspond to cross section (magnification x2000, from top to bottom, respectively) and right-hand pictures to the top surface view (magnification x5000) of the membranes.

Figure 7. SEM images of (**a)** 0.5, **(b)** 1.0, **(c)** 2.5 wt.% ZIF-8/CA MMMs. Left-hand pictures correspond to cross section (magnification x2000, from top to bottom, respectively) and right-hand pictures to the top surface (magnification x5000, from top to bottom, respectively) view of the membranes.

Figure 8. Single gas permeability values of **(a)** CO_2, **(b)** CH_4 and **(c)** N_2 of the CA-DMC membranes as a function of filler loading. Ideal selectivities for **(d)** CO_2/N_2 and **(e)** (CO_2/CH_4) gas pair mixtures. Full and void symbols and bars correspond to the data obtained with the fresh (full) and conditioned (void) membranes, respectively.

Figure 9. Robeson's upper bound for the **(a)** CO_2/CH_4 and **(b)** CO_2/N_2 permeability-selectivity trade-off.

Figure 10. Selectivity enhancement of CA-DMC based MMMs vs CO_2 permeability enhancement for **(a)** CO_2/N_2 and **(b)** CO_2/CH_4 separation, respectively.

Figure 11. F-index of the prepared CA-DMC MMMs for CO_2/CH_4 separation.

Table S1. Relevant literature found on the synthesis and characterization of CA membranes for different applications.

**Membrane (*)**	**Solvent (*)**	**Characterization Techniques (*)**	**Application**	**Reference**
PVA/CA	DMSO	SEM, contact angle, XRD, mechanical tests	Pervaporation	Zhou et al. [1]
PEG/CA	Acetone	FTIR, XRD, SEM, AFM, roughness, contact angle	Water treatment	Ahmad et al. [2]
MCC/CA	DMF	XRD, FTIR, TGA, SEM, contact angle, porosity, mechanical tests	Water treatment	Abdullah et al. [3]
β-CD/CA-PANI	DMF: DCM: MeOH (3:2:1 v/v)	SEM, FTIR, XRD, TGA, zeta potential	Adsorption	Ali et al. [4]
MMT/CA	NMP	ATR-FTIR, XPS, AFM, XRD, FESEM, porosity, water contact angle	Ion exchange	Millare et al. [5]
MC/CA	Acetone/DMAc	SEM, FTIR, OCA, mechanical test, WHC	Biological applications	Naeem et al. [6]
ZnO/CA	DMF	SEM, porosity, FTIR, mechanical tests, contact angle, XRD, zeta potential	Ultrafiltration	Asiri et al. [7]

Membrane (*)	Solvent (*)	Characterization Techniques (*)	Application	Reference
Cu-MOF-GO/CA	THF	SEM, FTIR, contact angle, mechanical tests	Environmental Remediation	Rehman et al. [8]
CSNps-ZEO/CA	Acetic acid/acetone (7:3 v/v)	SEM, ATR-FTIR, DSC, porosity, water permeability, mechanical tests, contact angle, antibacterial assays	Food packaging	Nazari et al. [9]
CA	Cyrene	FTIR, SEM, contact angle, nanoindentation, TGA, swelling,	Organic solvent nanofiltration (OSNF)	Abdellah et al. [10]
CA 50,000 Mn (same as this work)	THF	Salt permeability, salt sorption, water uptake, DSC	$LiCl/MgCl_2$ selective membranes	Irving et al. [11]

(*) AFM: atomic forced microscopy; CSNPs: CS nanoparticles; Cu-MOF: Copper (II)-1,3,5-benzene tricarboxylic acid; DCM: dichloromethane; DMAc: N-N, dimethyl acetamide; DMF: dimethylformamide; DMSO: dimethyl sulfoxide; DSC: Differential Scanning Calorimetry; FESEM: Field Emission Scanning Electron Microscopy; FTIR: Fourier Transform Infrared Spectroscopy; GO: Graphene Oxide; MC: microbial cellulose; MCC: microcrystalline cellulose; MeOH: methanol; MMT: montmorillonite; NMP: N-methyl-2-pyrrolidone; OCA: optical contact angle; PAN_I: polyaniline; PEG: polyethylene glycol; PVA: poly(vinyl) alcohol; SEM: Scanning Electron Microscopy; TGA: Thermal Gravimetric Analyses; THF: Tetrahydrofuran; WHC: Water Holding Capacity; XPS: X-ray Photoelectron Spectroscopy; XRD: X-Ray Diffraction; ZEO: *Ziziphora clinopodioides* essential oils; ZnO: Zinc oxide; β-CD: β-cyclodextrin

Table S2. Hansen Solubility Parameters (HSPs) of different fossil-fuel-based solvents used in the industry and some green solvents compared to cellulose acetate (CA).

**Material**	**δ_D (MPa$^{0.5}$)**	**δ_P (MPa$^{0.5}$)**	**δ_H (MPa$^{0.5}$)**	**δ_T (MPa$^{0.5}$)**	**R_a(-CA)**
Water	15.1	20.4	16.5	30.3	11.62
Acetone	15.5	3.9	9.7	18.7	10.13
Chloroform	17.8	3.1	5.7	19.0	10.64
Dichloromethane (DCM)	17	7.3	7.1	19.8	6.74
Tetrahydrofuran (THF)	16.8	5.7	8	19.5	7.78
Dimethylformamide (DMF)	17.4	13.7	11.3	24.8	2.12
N,N-dimethylacetamide (DMAc)	16.8	11.5	9.4	22.4	3.26
Dimethyl carbonate (DMC)	15.5	3.9	9.7	18.7	10.13
Diethyl carbonate (DEC)	15.1	3.5	6.3	16.7	11.74
Cellulose Acetate (CA)	18.2	12.4	10.8	24.5	-
ZIF-8 (2MI) (*)	18.8	10.7	9.7	23.7	2.35
CO_2	15.7	6.3	5.7	17.85	9.39
N_2	11.9	0	0	11.9	20.72
CH_4	14.0	0	0	14.0	18.47

(*) 2MI is the functional group in ZIF-8 responsible for interactions with other components [12,13].

References

(1) Zhou, K.; Zhang, Q. G.; Han, G. L.; Zhu, A. M.; Liu, Q. L. Pervaporation of Water-Ethanol and Methanol-MTBE Mixtures Using Poly (Vinyl Alcohol)/Cellulose Acetate Blended Membranes. *J. Membr. Sci.* **2013**, *448*, 93–101.

(2) Ahmad, A.; Jamshed, F.; Riaz, T.; Sabad-E-Gul; Waheed, S.; Sabir, A.; Alanezi, A. A.; Adrees, M.; Jamil, T. Self-Sterilized Composite Membranes of Cellulose Acetate/Polyethylene Glycol for Water Desalination. *Carbohydr. Polym.* **2016**, *149*, 207–216.

(3) Abdullah, R.; Astira, D.; Zulfiani, U.; Widyanto, A. R.; Hidayat, A. R. P.; Sulistiono, D. O.; Rahmawati, Z.; Gunawan, T.; Kusumawati, Y.; Othman, M. H. D.; Fansuri, H. Fabrication of Composite Membrane with Microcrystalline Cellulose from Lignocellulosic Biomass as Filler on Cellulose Acetate Based Membrane for Water Containing Methylene Blue Treatment. *Bioresour. Technol. Rep.* **2024**, *25*, 101728.

(4) Ali, A. S. M.; El-Aassar, M. R.; Hashem, F. S.; Moussa, N. A. Surface Modified of Cellulose Acetate Electrospun Nanofibers by Polyaniline/β-Cyclodextrin Composite for Removal of Cationic Dye from Aqueous Medium. *Fibers Polym.* **2019**, *20* (10), 2057–2069.

(5) Ang, M. B. M. Y.; Devanadera, K. P. O.; Duena, A. N. R.; Luo, Z. Y.; Chiao, Y. H.; Millare, J. C.; Aquino, R. R.; Huang, S. H.; Lee, K. R. Modifying Cellulose Acetate Mixed-Matrix Membranes for Improved Oil-Water Separation: Comparison between Sodium and Organo-Montmorillonite as Particle Additives. *Membranes* **2021**, *11(2)*, 80-92.

(6) Naeem, M. A.; Siddiqui, Q.; Leroy, A.; Khan, M. R.; Wei, Q. The Production and Characterization of Microbial Cellulose–Electrospun Membrane Hybrid Nano-Fabrics. *J. Ind. Text.* **2021**, *51* (3), 380–395.

(7) Asiri, A. M.; Petrosino, F.; Pugliese, V.; Khan, S. B.; Alamry, K. A.; Alfifi, S. Y.; Marwani, H. M.; Alotaibi, M. M.; Algieri, C.; Chakraborty, S. Synthesis and Characterization of Blended Cellulose Acetate Membranes. *Polymers* **2022**, *14*, 4-21.

(8) Rehman, A.; Jahan, Z.; Sher, F.; Noor, T.; Khan Niazi, M. B.; Akram, M. A.; Sher, E. K. Cellulose Acetate Based Sustainable Nanostructured Membranes for Environmental Remediation. *Chemosphere* **2022**, *307*, 135736.

(9) Nazari, M.; Majdi, H.; Gholizadeh, P.; Kafil, H. S.; Hamishehkar, H.; Zarchi, A. A. K.; Khoddami, A. An Eco-Friendly Chitosan/Cellulose Acetate Hybrid Nanostructure Containing Ziziphora Clinopodioides Essential Oils for Active Food Packaging Applications. *Int. J. Biol. Macromol.* **2023**, *235*, 123885.

(10) Abdellah, M. H.; Oviedo, C.; Szekely, G. Controlling the Degree of Acetylation in Cellulose-Based Nanofiltration Membranes for Enhanced Solvent Resistance. J. Membr. Sci. 2023, 687, 122040.

(11) Irving, P. R.; Reimund, K. K.; Zofchak, E. S.; Marioni, N.; Freeman, B. D.; Ganesan, V. Cellulose Acetate Membranes Exhibit Exceptional Monovalent to Divalent Cation Selectivities. *J. Membr. Sci.* **2024**, *706*, 122892.

(12) Paseta, L.; Potier, G.; Abbott, S.; Coronas, J. Using Hansen Solubility Parameters to Study the Encapsulation of Caffeine in MOFs. *Org. Biomol. Chem.* **2015**, *13* (6), 1724–1731.

(13) Casado-Coterillo, C.; Fernández-Barquín, A.; Zornoza, B.; Téllez, C.; Coronas, J.; Irabien, Á. Synthesis and Characterisation of MOF/Ionic Liquid/Chitosan Mixed Matrix Membranes for CO_2/N_2 Separation. *RSC Adv.* **2015**, *5* (124), 102350–102361.
